# Crosstalk between the mitochondrial fission protein, Drp1, and the cell cycle is identified across various cancer types and can impact survival of epithelial ovarian cancer patients

**DOI:** 10.18632/oncotarget.11047

**Published:** 2016-08-04

**Authors:** Deepak Kumar Tanwar, Danitra J. Parker, Priyanka Gupta, Brian Spurlock, Ronald D. Alvarez, Malay Kumar Basu, Kasturi Mitra

**Affiliations:** ^1^ Department of Pathology, University of Alabama at Birmingham, Birmingham, AL, USA; ^2^ Department of Genetics, University of Alabama at Birmingham, Birmingham, AL, USA; ^3^ Department of Obstetrics and Gynecology, University of Alabama at Birmingham, Birmingham, AL, USA

**Keywords:** Drp1, mitochondria, cell cycle, cancer, genome analyses

## Abstract

Mitochondrial metabolic reprogramming is a hallmark of tumorigenesis. Although mitochondrial function can impact cell cycle regulation it has been an understudied area in cancer research. Our study highlights a specific involvement of mitochondria in cell cycle regulation across cancer types. The mitochondrial fission process, which is regulated at the core by Drp1, impacts various cellular functions. Drp1 has been implicated in various cancer types with no common mechanism reported. Our Drp1-directed large-scale analyses of the publically available cancer genomes reveal a robust correlation of Drp1 with cell-cycle genes in 29 of the 31 cancer types examined. Hypothesis driven investigation on epithelial ovarian cancer (EOC) revealed that Drp1 co-expresses specifically with the cell-cycle module responsible for mitotic transition. Repression of Drp1 in EOC cells can specifically attenuate mitotic transition, establishing a potential casual role of Drp1 in mitotic transition. Interestingly, Drp1-Cell-Cycle co-expression module is specifically detected in primary epithelial ovarian tumors that robustly responded to chemotherapy, suggesting that Drp1 driven mitosis may underlie chemo-sensitivity of the primary tumors. Analyses of matched primary and relapsed EOC samples revealed a Drp1-based-gene-expression-signature that could identify patients with poor survival probabilities from their primary tumors. Our results imply that around 60% of platinum-sensitive EOC patients undergoing relapse show poor survival, potentially due to further activation of a mitochondria driven cell-cycle regime in their recurrent disease. We speculate that this patient group could possibly benefit from mitochondria directed therapies that are being currently evaluated at various levels, thus enabling targeted or personalized therapy based cancer management.

## INTRODUCTION

Specific alteration of mitochondrial and cellular metabolism is one of the hallmarks of tumorigenicity [1], with certain driver mutations identified in mitochondrial metabolic components [2]. Currently, efforts are underway to develop mitochondria based cancer therapeutics, with some at the level of clinical trials [3]. Mitochondria are multifaceted organelles that are at the center stage of energetics/metabolism but can also take part in cellular redox and calcium balance, lipid modification, and cell death [4]. Mitochondria can also take part in active control of cell cycle, which has been hypothesized to maintain deregulated cell proliferation in tumors [5]. Part of the cell cycle control by mitochondria is mediated by proteins that promote either fission or fusion between individual mitochondria that impact their functionality [6]. The crosstalk between the mitochondrial fission/fusion proteins and oncogenic regulators is recently being appreciated [7]. Dynamin Related Protein 1 (Drp1) constitutes the core component of the mitochondrial fission machinery and thus regulates mitochondrial structure-function relationship [8]. To be able to cause mitochondrial fission Drp1 gets recruited to mitochondria from the cytosol by either Fis1 or MFF1, two mitochondrial proteins [6]. The Drp1 activity can also be modulated by Mid49/51 to govern mitochondrial fission [9]. On the other hand, Drp1 driven mitochondrial fission is opposed by mitochondrial fusion governed primarily by MFN1/2 and Opa1 [6].

The gene coding for Drp1, DNM1L, can be regulated at the level of transcription [10–13], while the function of the Drp1 protein can be regulated by various post-translational modifications [14]. Loss of Drp1 is embryonic lethal in various model organisms [8] and Drp1 has been found to be critical for proper functioning of tissues like brain [15], heart [16] and ovary [17] in mouse models. At the cellular level, Drp1 has been implicated in various processes like apoptosis [18], mitophagy [19], metabolism/energetics [20], immune response [21, 22], cell proliferation and differentiation [23, 24] as well as cell transformation [25]. Drp1 activity has been found to be integrated into cell cycle control; cell cycle regulators modulate Drp1 which in turn regulates other cell cycle molecules [5]. The existing model suggests that Drp1 activity is elevated during active mitosis and is lowered before DNA synthesis. Drp1 has been functionally or molecularly linked to the major cyclins, Cyclin B [26, 27], Cyclin E [24–28] and Cyclin D [29]. Previous studies from our laboratory and others demonstrate that Drp1 driven mitochondrial fission is critical for regulation of cell proliferation in a *Drosophila* model system, as well as in mammalian cells [5]. Nonetheless, the involvement of Drp1 as well as other mitochondrial fission/fusion proteins in cell cycle remains an understudied area. Based on the current limited findings, we speculated that regulation of cell cycle by Drp1 may be critical in maintaining tumorigenic cell proliferation.

Drp1 has already been implicated in the development of cancer of the breast, lung, skin and brain, but no common underlying mechanism has been reported in these findings: Drp1 has been proposed to alter mitochondrial energetics and cellular metabolism to sustain tumor development in melanoma [25], regulate stem cell maintenance in glioblastoma [30], promote metastasis in breast cancer [31] while maintain cell proliferation in lung cancer cells [32]. Given that Drp1 has been shown to be involved in myriad of cellular processes (mentioned before), investigation of the common or unique cellular modules affected by Drp1 driven mitochondrial fission is pertinent in the context of tumorigenesis. Here, we undertook an exploratory approach to identify the Drp1 related cellular functional modules across various cancer types. We performed large-scale genomic analyses from publically available cancer genome data and found that Drp1 expression is robustly associated with the expression of cell cycle genes in almost all the cancer types examined. We have previously demonstrated a role of Drp1 in regulating cell proliferation of the ovarian epithelial cell layer in *Drosophila* [23]. Thus, here we focused on more detailed and stringent investigation of the role of Drp1 in epithelial ovarian cancer (EOC) that is the most prevalent form of ovarian cancer [33], where specific cell cycle regulation is perturbed in more than 80% of the patients [34]. Our results demonstrate for the first time that Drp1 driven regulation of mitosis potentially supports cell proliferation in the development of primary and relapsed EOC in distinct groups of patients. Moreover, we found that a Drp1-based-gene-expression-signature when employed on the primary tumors can identify a specific group of EOC patients with poor survival. We speculate that this patient group could possibly benefit from mitochondria directed chemotherapeutics [3].

## RESULTS

### “Cell-Cycle” identified as a major Drp1 co-expression module across tumor types

**Figure 1:**
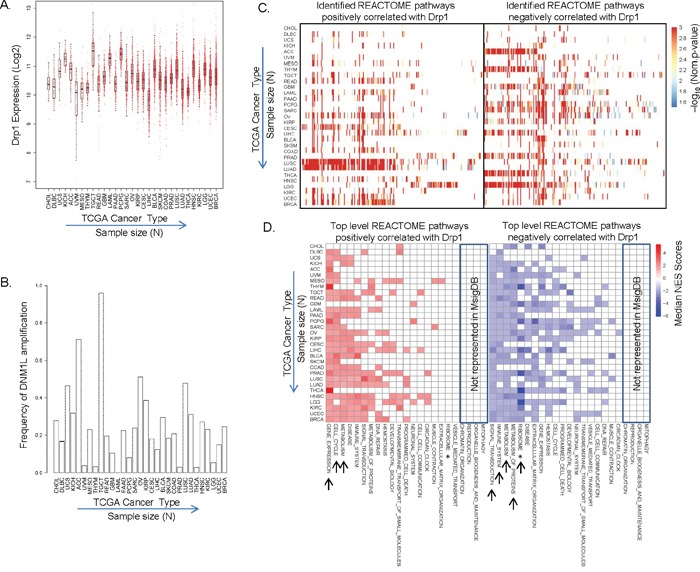
Identification of Drp1 co/anti-expression modules across TCGA tumor types **A.** Box plot of Drp1 expression (RNA-seq) in tumor tissues across the TCGA cancer types. **B.** Bar plot representing patient frequency with more than one copy of DNM1L gene (based on GISTIC scores) in tumor tissues across the TCGA cancer types. **C.** Heat map of the functional REACTOME pathways correlating positively (left) or negatively (right) with Drp1 expression (X axes) in 31 TCGA cancer types (Y axes), as identified by GSEA analyses of the Drp1 correlating genes. Heat map represents negative log converted *p* values of the individual REACTOME pathways (within the cut of *p* and *q* ≤0.05). **D.** Heat map of the top level REACTOME pathways (X axes) depicted in C. Heat map represents median NES scores of the identified pathways (see Supplementary Figure S2). Top-level pathways present in REACTOME but not in MsigDB are boxed. * depicts re-assigned “Ribosome” pathway. Arrows indicate the pathways common in majority of the cancer types.

Mitochondria are multifunctional organelles and are programmed to perform different roles depending on the tissue and cell type [4]. Therefore, the various reported roles of Drp1 driven mitochondrial fission can be alluded to distinct involvement of Drp1 driven mitochondrial fission in various tissues. However, no large-scale studies have been performed to assess the role of Drp1 across various normal or cancer tissues. The availability of genomic data from 31 different cancer types in The Cancer Genome Atlas (TCGA) provides the opportunity to analyze and predict any common or unique role of Drp1 driven mitochondrial fission in various tumor types. Therefore, we aimed to identify any Drp1 gene co/anti-expression modules in the primary tumors of TCGA (see Supplementary Table 1A for the description of the TCGA cancer types). We began with analyses of normalized Drp1 expression (RNAseq) across the TCGA primary tumor types. Drp1 expression varies across the various TCGA cancer types, with median Drp1 expression being maximum in TGCT and minimum in LIHC (Figure 1A, Supplementary Table 1A). To investigate if elevated Drp1 expression is a result of somatic copy number amplification (SCNA) we correlated Drp1 expression with the GISTIC scores (Genomic Identification of Significant Targets in Cancer [35]) of the Drp1 gene, DNM1L, across the cancer types. Various levels of SCNA were noted for the DNM1L gene in various cancer types, with almost 100% of TGCT patients presenting with DNM1L gene amplification (Figure 1B). Linear regression analyses between DNM1L GISTIC scores and Drp1 expression at the corresponding GISTIC levels revealed a significant correlation between the two parameters (Supplementary Figure S1A). Next, we performed Pearson's correlation analyses to identify the Drp1 correlated genes, and then employed the functional class scoring method called Gene Set Enrichment Analyses (GSEA) on the significant Drp1 correlated genes (*p*≤0.05) to identify the Drp1 correlated pathways from the hand curated REACTOME pathways in the MsigDB database (Supplementary Table 2). Expectedly, the number of statistically significant Drp1 correlated genes was proportional to the sample size (N) of the cancer types (Supplementary Figure S1B). Nonetheless, Drp1 correlated pathways (GSEA *p* and *q*≤0.05) were identified in each of the 31 cancer types: some cancer types exhibit greater abundance (indicated by converted *p* values) of pathways positively correlating with Drp1 (ex: LUSC, LUAD, CESC, READ) while others exhibit abundance of pathways negatively correlating with Drp1 (ex: ACC, THYM, GBM, PCPG, SARC, PRAD, THCA) (Figure 1C). Next we mapped the identified Drp1 correlated REACTOME pathways to their corresponding top level REACTOME categories using the pathway relationship and obtained the median GSEA Normalized Enrichment Score (NES) for each top level REACTOME category. We found that Drp1 positively correlates with “Gene Expression”, “Cell Cycle” and “Metabolism” and negatively with “Signal Transduction”, “Immune System”, “Metabolism”, “Metabolism of Proteins” in majority of the cancer types (Figure 1D. Supplementary Figure S2). In majority of the cancer types Drp1 also negatively correlated strongly with ribosomal genes that led to the identification of various REACTOME pathways that we reassigned to a new top level category called “Ribosome” (see methods). Notably, Drp1 uniquely correlated with the “Neuronal System” pathway in the gliomas (GBM and LGG) and “Muscle Contraction” in sacrcoma (SARC) and mesothelioma (MESO). Also, Drp1 correlated with “DNA Repair” pathways in distinct cancer types (BRCA, UCEC, LUAD, OV etc). Importantly, Drp1 correlated positively or negatively with “Programmed Cell Death” pathway in distinct cancer types, consistent with its reported involvement in apoptosis [18]. The top-level pathways where Drp1 correlated genes were not detected (ex: “Mitophagy”) could be due to lack of representation of those pathways in the hand curated MsigDB (Figure 1D).

Taken together, our Drp1 based analyses of the 31 TCGA tumor types highlighted the cellular functions that are related to Drp1 across various cancer types with varying degree of Drp1 expression and DNM1L gene amplification. Of the various functions Drp1 has been reported to be involved in, Cell Cycle was identified as a major Drp1 correlating module widely across cancer types; co-expression in 16 cancer, anti-expression in 5 cancer, co/anti expression in 8 cancer (could be due to distinct gene sets in co and anti expression categories), no correlation in 2 cancer (likely due to low sample size).

### Drp1 co-expresses predominantly with mitosis genes, independent of gene duplication events, and supports cell proliferation in epithelial ovarian cancer

**Figure 2:**
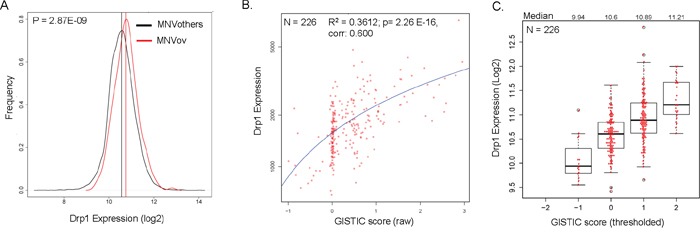
Elevated expression of Drp1 due to gene duplication in epithelial ovarian tumors **A.** Density plots depicting the frequency distribution of Drp1 expression in the primary epithelial ovarian tumors (red) in comparison to all other primary tumors of TCGA (black). **B.** Linear regression analysis of Drp1 expression (plotted on a log scale) and raw GISTIC scores in individual EOC patients (red dots). Regression coefficient (R^2^), *p* value and correlation mentioned. **C.** Box-plot of Drp1 expression at various thresholded GISTIC levels in EOC patients; median Drp1 expression values mentioned on top. N denotes sample size.

We have previously shown that perturbation of Drp1 activity causes aberration in cell proliferation in the ovarian epithelial cell layer of *Drosophila* [23, 36]. Based on this and our current finding (Figure 1) we hypothesized that elevated Drp1 levels in epithelial ovarian cancer (EOC) would be necessary for promoting cell proliferation in the EOC patients, where a specific cell cycle pathway was found to be perturbed in greater than 80% of the patients [34]. We found that the Median Normalized expression Value (RNA-seq) of Drp1 in the TCGA-EOC tumors (MNVov) is modestly but significantly higher (14.6 %) than that of all other cancers pooled together (MNVothers) (Figure 2A). DNM1L (cytogenetic location: 12p11.21), resides in one of the regional amplified genomic regions identified in the TCGA-EOC patients [37]. Indeed, we found the DNM1L gene amplification was detected in around 50% of the EOC patients (Figure 1B, Ov). Linear regression analyses between DNM1L GISTIC scores and Drp1 expression at the corresponding GISTIC levels revealed a strong correlation between the two parameters in individual EOC patients (Figure 2B). The expected fold increase in Drp1 expression at increasing GISTIC levels in the EOC tumors confirm that the DNM1L gene is amplified in the EOC patients leading to elevated expression of Drp1 (Figure 2C). Taken together, we concluded that Drp1 expression is elevated due to DNM1L duplication in EOC tumors.

**Figure 3:**
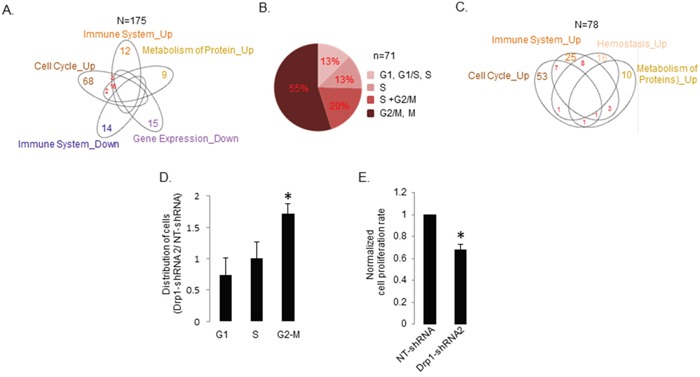
Drp1 co-expresses with cell cycle genes, independent of gene duplication events, and supports proliferation of the EOC cells **A.** Venn diagram depicting the overlap in the leading edge genes of the REACTOME top-level pathways correlating positively (Up) or negatively (Down) with Drp1 expression in the primary EOC tumors of TCGA. Numbers signify the total number of leading edge genes. **B.** Pie chart representing distribution of Drp1 co-expressing REACTOME Cell Cycle leading edge genes in various phases of cell cycle (G1 phase, G1/S transition, S phase, G2/M transition and M (mitosis)). The Drp1 co-expressing genes involved in proteasomal function that are assigned to all cell cycle phases were excluded in this classification. **C.** Venn diagram depicting the overlap in the leading edge genes of the REACTOME top-level pathways correlating positively (Up) with Drp1 expression in the TCGA EOC patients with no amplification of DNM1L gene in their primary tumors; no statistically significant negatively correlated pathways were detected. Numbers signify the total number of leading edge genes. **D.** Bar plot showing the distribution of cells in G1, S and G2-M phases in A2780 cell line stably expressing Drp1shRNA2 as normalized by the distribution of A2780 cell line stably expressing a non-targeting (NT) shRNA. **E.** Bar plot showing the cell proliferation rate of A2780 cell line stably expressing Drp1shRNA2 as normalized by that of A2780 cell line stably expressing a non-targeting (NT) shRNA. n denotes number of genes; N denotes number of patients. * denotes *p* value < 0.05 in Student's t-test.

Next, we wanted to perform more detailed and stringent Drp1 co-expression analyses to understand the Drp1-Cell-Cycle co-expression module in the TCGA EOC samples. To maintain robustness of our conclusion here we only included samples where the normalized Drp1 expression values lie within “one” Median Absolute Deviation (MAD) [34] (median +/−1 MAD) and only those genes with expression levels above “zero” in the MAD1 patient group. Such filtering allowed expression analyses of about 15,000 genes(n) from 175 tumors (N), which represent our TCGA-EOC data set presented here, unless otherwise stated. GSEA analyses of the statistically significant (*p*≤0.05) Drp1 correlated gene set (Supplementary Table 3A) in the Drp1-MAD1 EOC patient group was performed. Of the various REACTOME pathways identified (Supplementary Figure S3A) we considered only the top 20 (based on NES, Supplementary Figure S4) significant pathways (*p* and *q*≤0.05) and mapped them to their top-level categories. Such stringent GSEA analyses in the Drp1-MAD1 group confirmed “Cell Cycle” as the predominant top-level category that positively correlated with Drp1 expression in the REACTOME (Figure 3A, Supplementary Table 4A) as well as in the KEGG pathways (Supplementary Table 4B). Moreover, the identified “Immune System” and “Metabolism of Protein” pathways had their leading-edge gene sets (contributing mostly to the identification of a pathway) significantly overlapping with that of the “Cell Cycle” category (Figure 3A, Supplementary Table 5). However, we did not detect “Gene Expression” or “Metabolism” categories positively correlating with Drp1 in the Drp1-MAD1 group. Instead, Gene Expression was identified as a negatively Drp1 correlating pathway along with “Immune System” (with distinct genes from the positively correlated “Immune System”) (Figure 3A, Supplementary Table 5).

Further classification of the Drp1 co-expressing cell cycle genes according to their involvement in various cell cycle phases revealed that 55% of the Drp1 co-expressed cell cycle genes are specifically involved in mitotic transition (Figure 3B, Supplementary Table 5). We noted that although we detected Drp1-coexpression with various mitotic genes that are transcriptionally controlled by the FoxM1 transcription factor [34], we did not identify FoxM1 itself due to its absence in the hand curated MsigDB. However, our targeted investigation revealed that Drp1 indeed co-expressed with FoxM1 and its downstream mitotic genes (Supplementary Figure S3C) previously shown to be perturbed in majority of the TCGA-EOC patients [34]. Importantly, Drp1 also predominantly co-expressed with cell cycle genes even in the tumors with no SCNA of the DNM1L gene (GISTIC score 0) (Figure 3C, Supplementary Figure S3B), thus ruling out any confounding effect of gene co-amplification on Drp1 co-expression. We noted that Drp1 expression did not correlate with that of KRas in tumors with no DNM1L SCNA, although the Drp1 correlation with FoxM1 was maintained (Supplementary Figure S3D). Given the KRas gene (12p12.1) resides in between DNM1L (Drp1 gene) (12p11.21) and FoxM1 gene (12p13), we reasoned that any confounding effect of regional epigenetic regulation is highly unlikely, thus hinting towards a potential significance of the correlation of Drp1 with FoxM1 involved in mitosis. Drp1-Cell-Cycle gene co-expression was also identified as the predominant category in tumors with DNM1L SCNA (GISTIC score≥1) as well as in tumors outside the Drp1-MAD1 range (not shown).

Next, we sought to investigate whether the cell cycle association observed with Drp1 also occurs for other fission and/or fusion factors of mitochondria. Towards this end, we performed pairwise comparison of the genome wide correlation profile of Drp1 expression and each of the other fission proteins, namely Fis1, Mid49, Mid51, Mff or fusion proteins namely Mfn1, Mfn2, Opa1. Our approach involved a primary gene based comparison followed by a secondary pathway based comparison. Given, gene duplication may be a confounding factor, we first identified the patients with no gene amplification (GISTIC 0) for each gene of interest and then identified the common patients between Drp1 GISTIC 0 and that of the other gene of interest in each pairwise comparison (Supplementary Figure S5). Then, we identified the genes that are commonly correlated (*p*≤0.05) between Drp1 and the other gene of interest and performed GSEA of the common genes using correlation values with Drp1 and the other gene of interest (Supplementary Figure S6). Finally, we computed the overlap between the identified (not top level) pathways and compared the leading edge genes of any identified cell cycle categories (Supplementary Table 6). From these thorough stringent analyses we found that the genome wide correlation profile of Drp1 matches overwhelmingly with Mff at every level of analyses and common cell cycle pathways were identified only in the pairwise comparison of Drp1 and Mff (Table 1); 50 leading edge Cell Cycle genes comprise the Drp1:MFF overlapping gene set with only a small fraction of this gene set being present in the correlation profile of Fis1, Mfn1, Mfn2 and Opa1 (Supplementary Table 7). Although MIEF1 had some significantly correlated gene overlap with Drp1, no significant pathways were identified in GSEA. The overlapping gene set between Drp1 and Fis1 was not statistically significant and did not identify any overlapping pathway, although Fis1 correlation module itself identified a distinct set of pathways (Supplementary Figure S5, Supplementary Table 6). Importantly, we did not identify any negative correlation with cell cycle and any of the mitochondrial fusion proteins. However, Drp1 and Mfn2 had a complete overlap of all the 12 identified negatively correlating pathways, which predominantly consisted of “Ribosome” and “Gene Expression” categories (Supplementary Figure S5, Supplementary Table 6).

**Table 1:** Comparison of genome wide correlation profile of Drp1 expression with that of the other mitochondrial fission and fusion proteins in TCGA EOC patients

Gene Based Analysis								Pathway Based Analysis		
Gene1	Gene2	Correlation Category	No. of Genes Correlatingwith Gene1(p <= 0.05)[x1]	No. of Genes Correlating with Gene2(p <= 0.05)[x2]	Overlap[y=x1:x2]	Fisher ExactTest (p-value)	Jaccard Index forgeneoverlap	Common cell cyclepathways	DNM1L:MFFcorrelatedcell cycle leadingedge genes[z]	% correlated cell cycleleading edge genes[z/y *100]
DNM1L	FIS1	Positive	2934	2735	125	1	0.022546898	0	7	5.6
DNM1L	MFF	Positive	2677	2488	614	1.52665E-15	0.134915403	9	50	8.143322476
DNM1L	MFN1	Positive	907	689	47	0.343676161	0.030342156	0	3	6.382978723
DNM1L	MFN2	Positive	1836	1687	291	3.90929E-08	0.090037129	0	5	1.718213058
DNM1L	MIEF1	Positive	1151	1170	238	5.48548E-44	0.114258281	0	0	0
DNM1L	MIEF2	Positive	766	731	6	1	0.004024145	0	0	0
DNM1L	OPA1	Positive	973	1194	178	1.43616E-24	0.089492207	0	2	1.123595506
DNM1L	FIS1	Negative	1456	3711	116	1	0.022965749	0	0	0
DNM1L	MFF	Negative	1360	2647	394	1.44191E-22	0.10905065	0	0	0
DNM1L	MFN1	Negative	480	886	45	0.004192997	0.034065102	0	0	0
DNM1L	MFN2	Negative	747	1386	134	1.27922E-12	0.067033517	0	0	0
DNM1L	MIEF1	Negative	455	630	62	2.01866E-15	0.060606061	0	0	0
DNM1L	MIEF2	Negative	327	642	7	0.992945943	0.007276507	0	0	0
DNM1L	OPA1	Negative	296	955	48	1.07603E-08	0.039900249	0	0	0

In summary, our exploratory analyses of the TCGA-EOC genome reveal that Drp1 (and Mff) co-expresses with cell cycle genes, specifically the ones promoting mitotic transition of cell cycle. These observations are consistent with the previous findings that Drp1 function is elevated specifically during mitotic transition of the cell cycle [26]. Next, we determined if Drp1 is required for mitotic transition during the proliferation of EOC cells. Here, we downregulated Drp1 using 2 distinct shRNAs in the widely used A2780 ovarian cancer cell line and generated Drp1 knocked down stable A2780 lines (Supplementary Figure S7A). Since Drp1shRNA1 caused certain degree of cell death we chose to use the stable line expressing Drp1shRNA2 for our further experimentations. To investigate if Drp1 knockdown can prevent mitotic transition we performed flow cytometry analyses of the DNA content. Drp1 knocked down stable A2780 line (in sub-confluent cultures) had significantly greater number of cells in the G2-M phase in comparison to the A2780 line expressing non-targeting (NT) shRNAs, indicating Drp1 repression attenuates mitotic transition (Figure 3D). To rule out the effect of Drp1 repression is not due to a secondary compensatory response we wanted to confirm these effects of Drp1 repression in transiently knockdown cells; we chose the commonly used ovarian cancer cell lines, A2780 and SKOV3. We observed similar attenuation of mitosis with transient transfection of plasmids expressing both Drp1shRNAs in the A2780 line (Supplementary Figure S7B, S7C); note the degree of the detected mitotic attenuation varied significantly between experimental replicates presumably due to mitotic catastrophe [38]. Since the plasmid mediated expression of the Drp1shRNAs in the SKOV3 ovarian cancer cell line did not yield consistent knockdown of Drp1, we resorted to lentiviral mediated transduction of the Drp1shRNAs in the SKOV3 cells. Indeed, we found a similar trend of attenuation of mitosis with Drp1 knockdown in the SKOV3 cells (Supplementary Figure S7D, S7E). Attenuation of mitosis with Drp1 repression has also been observed in osteosarcoma cells [38] but not in lung cancer cells [32]. Furthermore, we found that Drp1shRNA2 stably expressing A2780 cells proliferated slowly in comparison to the A2780 cells expressing NTshRNAs, based on colony assay (Figure 3E). The transient Drp1 knockdown also showed similar results in both the cell lines, however, the associated cell death observed in the transient Drp1 knockdown cells remains a confounding factor in this case (Supplementary Figure S7F). Although, our experiments do not rule out involvement of Drp1 in other cell cycle stages, the data demonstrates a potential involvement of Drp1 in promoting mitotic transition and cell proliferation of ovarian cancer cells. These data provide validation for our genome analyses identifying cell cycle, specifically mitosis, as the most prominent Drp1 co-expression module in the primary EOC tumors. More experiments are necessary in establishing the exact mechanism of action for an active role of Drp1 in mitosis.

### Drp1-Cell-Cycle co-expression module is specifically detected in primary epithelial ovarian tumors that robustly respond to chemotherapy

**Figure 4:**
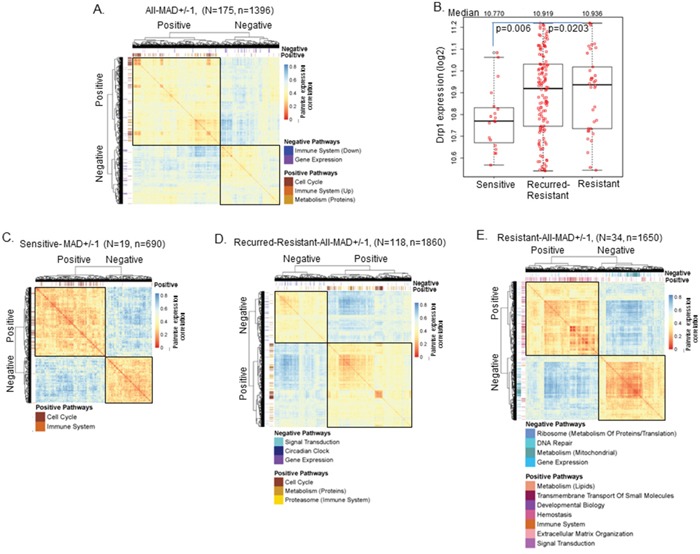
Drp1 expression and its correlation with cell cycle module vary with chemosensitivity **A.** Heat map and hierarchical clustering of the genes correlating positively or negatively with Drp1 expression in the primary EOC tumors of TCGA. Heat map represents gene to gene correlation matrix (in the form of distance) where the color scale depicts distance between the genes. The GSEA output is superimposed on the heatmap in the form of color-coded ticks representing the Drp1 correlating genes in various REACTOME pathways as labeled. **B.** Box plot of Drp1 expression in the primary tissues of sensitive, recurred-resistant and resistant EOC patients of TCGA. Median values of Drp1 expression mentioned on the top. *p* values signifies significance in permutation test. **C.** Heat map and hierarchical clustering (like in A) in Sensitive patients. **D.** Heat map and hierarchical clustering (like in A) in Recurred-Resistant patients. **E.** Heat map and hierarchical clustering (like in A) in Resistant patients. n denotes number of genes; N denotes number of patients. Extra description of the pathways in parenthesis is obtained from the REACTOME pathway hierarchy (see relevant supplementary table in each case).

Since the genes in the same biological phenomenon generally co-express together, we hypothesized that the Drp1 correlated genes would form distinct co-expressing clusters that would correspond to the REACTOME pathways identified by their GSEA analyses. Thus, we performed unsupervised hierarchical clustering analyses of the co-expression matrix of Drp1 co-related genes (p≤0.05). We calculated the all vs all correlation of the expression of these genes and clustered the correlation value after converting them into a Euclidian distance matrix where ‘zero’ distance corresponds to maximum gene correlation. Thereafter, we superimposed the REACTOME top-level category for each gene; Drp1 correlating genes identified in multiple REACTOME categories (Figure 3A) were assigned to unique categories with maximum correlation (see methods). We found that the Drp1 correlating genes clustered into two broad categories largely corresponding with the REACTOME pathways positively and negatively correlating with Drp1 (Figure 4A, boxed). More importantly, the Drp1 co-expressing cell cycle genes strongly clustered together within the positive gene cluster (Figure 4A, red clusters).

Next, we determined if Drp1 driven cell cycle regulation has any relationship to the patient chemoresistance status, since acquisition of chemoresistance to platinum based chemotherapy occurs in majority of the EOC patients [33]. First, we compared Drp1 expression between the patients with different clinical outcome to chemotherapy and disease progression [34] (see methods): disease free for more than 6 months (sensitive,11%), relapsing with resistant tumors after 6 months (recurred-resistant, 69%) and relapsing with resistant tumors within 6 months (resistant, 11%). Drp1 expression was found to be 7.7 % higher in the primary tumors of the recurred-resistant patients and 11.3 % higher in resistant patients in comparison to the sensitive patients (Figure 4B). We next performed GSEA and hierarchical clustering analyses of the co-expression matrix of the Drp1 correlated genes (as in Figure 4A) separately in the sensitive, recurred-resistant and resistant patient groups. In each group the identified two broad gene co-expression clusters largely corresponded with the identified REACTOME pathways that are positively and negatively correlated with Drp1. Interestingly, Drp1 correlated with distinct gene sets in each of the patient groups (Figure 4C-4E, Supplementary Figure 8A-8C, Supplementary Table 8). Importantly, Drp1-Cell Cycle co-expression clusters were identified only in the sensitive and recurred-resistant patients that responded robustly to chemotherapy but not in the resistant EOC patients, that did not respond as well to chemotherapy. In the resistant patients, the genes associated with Signal Transduction and Hemostasis were identified as predominant clusters positively correlating with Drp1, whereas, genes associated with mitochondrial metabolism was identified as a predominant cluster that negatively correlated with Drp1 only (Figure 4E). This data indicates the role of Drp1 is likely different in the resistant patients in comparison to the sensitive, or recurred resistant patients.

In summary, our detailed Drp1 based analyses of the primary tumors of the TCGA-EOC cohort confirm that Drp1 is co-expressed with cell cycle genes in chemosensitive tumors but not in the relatively chemoresistant primary tumors.

### Drp1 co-expresses with cell cycle genes in relapsed tumors of a specific group of epithelial ovarian cancer patients

**Figure 5:**
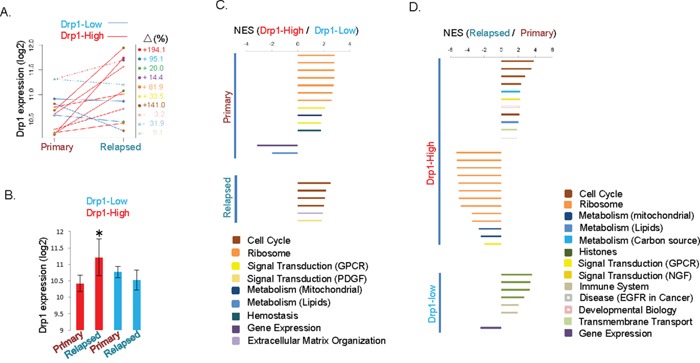
Drp1 co-expresses with cell cycle genes in relapsed tumors of a particular group of EOC patients **A.** Drp1 expression of various ICGC recurred-resistant EOC patients (color coded) in matched primary and relapsed disease. Lines connect the same patients. Patient with dashed line has been dropped from further analyses (please see relevant text in the Results section). The numbers on the right signify percentage increase (+) or decrease (−) in Drp1 expression in the relapsed samples in comparison to the primary. **B.** Barplots showing mean Drp1 expression in the primary and relapsed samples of Drp1-High and Drp1-Low patient groups of ICGC. * denotes p value < 0.05 in Student's t-test. **C.** Barplots showing the Normalized Enrichment Score (NES) of the pathways uniquely altered in the primary or relapsed samples between the Drp1-Hogh and Drp1-Low patient groups. The top-level REACTOME pathways are mentioned in the figure index. Extra description of the pathways in parenthesis is obtained from the pathway hierarchy (see relevant supplementary Table in each case), **D.** Barplots showing the Normalized Enrichment Score (NES) of the pathways uniquely altered in the Drp1-High or Drp1-Low patient groups between their relapsed and primary samples. The top-level REACTOME pathways are mentioned in the figure index. Extra description of the pathways in parenthesis is obtained from the REACTOME pathway hierarchy (see relevant supplementary Table in each case).

Majority of the EOC patients that initially respond to the treatment undergo relapse of the disease in a chemoresistant form [33]. Therefore, we investigated if Drp1 and its interplay with cell cycle are important in the relapsed chemoresistant disease. Here, we took advantage of the large-scale genomic data set published recently by the ICGC consortium towards understanding EOC chemoresistance [39]. We compared Drp1 expression (RNA-seq) from the paired primary sensitive tumor and relapsed resistant ascites samples of the recurred-resistant patient cohort (N=12, Supplementary Table 1 B-1C). We found that Drp1 expression in the relapsed samples was higher in comparison to the primary tissue (Supplementary Figure 9A). However, examination of individual patients revealed that 7 patients had increase in Drp1 levels in their relapsed samples (Figure 5A, Drp1-High) while 3 patients did not (Figure 5A, Drp1-Low); patient with two relapsed samples showing opposite trends of Drp1 expression (dotted lines in Figure 5A) was excluded from further analyses. Drp1 based re-grouping of patients revealed that Drp1-High group had 72% increase of Drp1 expression in their relapsed samples in comparison to their primary, while the decrease in the Drp1-Low group remains statistically insignificant likely due to low sample size (Figure 5B). To understand the significance, if any, of the elevated Drp1 expression in the relapsed samples we compared the Drp1-High and the Drp1-Low patient groups. Considering the low sample size we refrained from performing correlation analyses. Here, we compared the overall gene expression profile between Drp1-High and Drp1-Low groups to identify uniquely altered pathways in each group in their primary or relapsed samples. We performed GSEA analyses of the differentially expressed genes (≥2 fold) to identify functional pathways (*p* and *q*≤0.05). First, we identified the pathways that are differentially regulated uniquely in the primary or in the relapsed samples between the Drp1-High and Drp1-Low groups (strategy in Supplementary Figure 10A). We identified Cell Cycle pathway as predominantly boosted in the Drp1-High relapsed samples in comparison to that of Drp1-Low (Figure 5C, Supplementary Figure 11A-11B, Supplementary Table 9A-9B). This data, although indirectly, indicated a positive correlation between expression of Drp1 and cell cycle genes in the ICGC cohort, in consistent with the results from the TCGA cohort. Genes involved in “Extracellular Matrix Organization” and PDGF “Signal Transduction” pathways were also identified in this comparative analysis. Surprisingly, even the primary tumors of the Drp1-High group had a distinct gene expression profile compared to that of the Drp1-Low group: elevated expression of the “Ribosome” (reassigned, see methods), GPCR “Signal Transduction”, mitochondrial “Metabolism” and “Hemostasis” pathway components and lowered expression of “Gene Expression” and lipid “Metabolism” components in the Drp1-High group (Figure 5C). Next, we determined the distinct contribution of the Drp1-High and Drp1-Low groups in establishing these relative differences identified between the groups. Thus, we identified the pathways that are differentially regulated uniquely in the Drp1-High or Drp1-Low groups between their respective primary and relapsed samples (analysis strategy in Supplementary Figure 10B). Indeed, the Drp1-High EOC patients had elevated expression of cell cycle genes and lowered expression of genes involved in ribosomal function (reassigned, see methods), mitochondrial metabolism and GPCR signaling pathway in their relapsed samples in comparison to the primary samples (Figure 5D, Supplementary Figure 11C-11D, Supplementary Table 9C-9D). Importantly, this gene expression pattern was absent in the Drp1-Low groups where elevated expression of primarily the “Histone” genes (reassigned, see methods) was noted in the relapsed samples. Please note that although Cell Cycle does not emerge as a primary pathway that is altered between the primary and relapsed samples of the Drp1-low group, small sample size of this patient group presents an incomplete picture of the gene regulation in this group.

Relapse with chemoresistant disease may be triggered or maintained by cancer stem cells (CSCs) [40]. Aldh1A1 has been canonically used as marker for ovarian CSCs [41] and its function has also been shown to confer stem cell properties to ovarian CSCs [42]. We have previously demonstrated that transient repression of Drp1 elevates levels of Aldh1A1 in ovarian cancer cells (and other stem cell genes in other lineages) [36]. Consistently, here we find that Aldh1A1 was lowered in the relapse samples of the Drp1-High EOC patients that also have elevated levels of cell cycle genes (Supplementary Figure 9B), while the data remained inconclusive in the Drp1-Low group like due to its low sample size.

In summary, our analyses of the paired primary and relapsed EOC samples from the ICGC cohort demonstrate that patients that underwent an elevation of Drp1 expression in their relapsed disease (Drp1-High) had an associated increase in cell cycle genes that was not observed in the EOC patients that did not undergo the elevation of Drp1 in their relapsed disease (Drp1-Low).

### A Drp1-based-gene-expression-signature employed on primary tumors identifies recurred-resistant EOC patients with poor survival outcome

**Figure 6:**
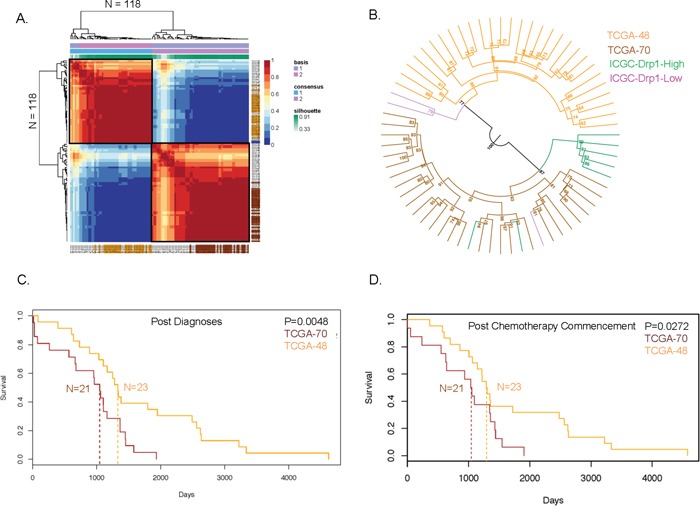
Drp1 based classification of the primary tumors of recurred-resistant EOC patients **A.** NMF consensus plot of 100 runs clustering the TCGA recurred-resistant EOC patients into TCGA-RR-70 (bottom right, red) and TCGA-RR-48 (top left, red) groups based on a Drp1-based-gene-expression-signature (basis). The color coded patients are the core of the TCGA-RR-70 (brown) or TCGA-RR-48 (orange) groups based on their weights. See Supplementary Table 10. **B.** Unrooted dendrogram showing hierarchical clustering of the TCGA-RR-70, TCGA-RR-48, ICGC-Drp1-High and ICGC-Drp1-Low patient groups. Bootstrap values on the nodes indicate branching confidence. **C.** Survival plot of the TCGA-RR-70 and TCGA-RR-48 patient groups after diagnoses of the disease as EOC. Dashed lines indicate mean survival probability. **D.** Survival plot of the TCGA-RR-70 and TCGA-RR-48 patient groups after commencement of chemotherapy. Dashed lines indicate mean survival probability. *p* values denote significance with a chi-square test.

We found that Drp1-High and Drp1-Low patients, with differential Drp1 regulation in their relapsed disease, exhibit distinct gene expression profiles in their primary tumors (Figure 5C). Therefore, we determined if the gene expression profile of the primary tumors of the Drp1-High and Drp1-Low ICGC groups match with that of distinct recurred-resistant patients from the larger TCGA-EOC cohort. Here, we considered the differential gene expression profile between the primary tumors of the ICGC-Drp1-High/Low groups as a Drp1-based-gene-expression-signature (≥2 fold difference, p≤0.05; n= 745, Supplementary Table 10B). This gene expression signature was used to perform Non-negative Matrix Factorization (NMF) of the primary tumors of the TCGA recurred-resistant EOC patients. The NMF algorithm divided the 118 patients into various clusters (Supplementary Figure 12A-12B), with maximum cophenetic (>0.95) and silhuoette score (1) for classification into 2 clusters of 70 (TCGA-RR-70) and 48 (TCGA-RR-48) patients (boxed in Figure 6A, Supplementary Figure 13A-13B). To determine which of these two TCGA groups correspond to the ICGC-Drp1-High group we performed hierarchical clustering of the TCGA and ICGC samples together, based on their Drp1-based-gene-expression-signature. Given the nature of the NMF clusters (Figure 6A, Supplementary Figure 13A, S13B), we identified the top 29 patients that formed the core of each cluster (Figure 6A, Supplementary Table 10A). Interestingly, the ICGC-Drp1-High group clustered with TCGA-RR-70 group (Figure 6B), which strongly suggested that the Drp1-based-gene-expression-signature profile is similar between these ICGC and TCGA groups. However, the correspondence of the ICGC-Drp1-low and the TCGA-RR-48 groups remained inconclusive, likely due to the low sample size of the ICGC-Drp1-Low group. Nonetheless, Drp1 expression level in the TCGA-RR-70 primary tissue was lower than that of TCGA-RR-48 (Supplementary Figure 14A), a trend that was observed between the ICGC-Drp1-High and Drp1-Low groups (Figure 5B). Consistently, the TCGA-RR-70 primary tissue had a weaker Drp1-Cell- Cycle co-expression cluster when compared to that of the TCGA-RR-48 group (not shown). GSEA analyses of the differentially expressed genes (≥2 fold) of the core TCGA-RR-70/48 and ICGC-Drp1-High/Low groups revealed that the core TCGA-RR-70 and the ICGC-Drp1-High groups have higher levels of genes involved in GPCR signaling and Hemostasis (Supplementary Figure 14B-14C). More importantly, the survival probability, specifically in the long term after diagnoses or the earliest chemotherapy, was significantly lower in the core TCGA-RR-70 group (median survival-35 months) in comparison to the core TCGA-RR-48 group (median survival-43 months) (Figure 6C, 6D). Given that the Drp1-based-gene-expression-signature of the TCGA-RR-70 group was similar to that of the ICGC-Drp1-High group (Figure 6B), our results raise the retrospective possibility that the TCGA-RR-70 group may have been more susceptible to activating the Drp1 based Cell Cycle module in their relapsed tumors, leading to a more aggressive relapse in comparison to the TCGA-RR-48 group.

In summary, our results suggest that a Drp1-based-gene-expression-signature can be potentially employed on the EOC primary tumors towards identification of patients who would be expected to have poor survival, potentially due to further activation of a Drp1 based cell cycle regime in their relapsed disease.

## DISCUSSION

Given mitochondria are the central players in cellular energetics/metabolism, detailed investigations are underway to understand the mechanistic involvement of mitochondria in cancer energetics/metabolism. In this regard, the involvement of the mitochondrial fission-fusion molecules in cancer has recently been appreciated and The mitochondrial fission protein, Drp1, has been directly implicated in various cancer types [25, 30–32]. Drp1 driven mitochondrial fission can be activated by various cancer signaling pathways involving PKA [43], AMPK [44] and EGFR-Ras [23–25, 45]. The Ras activated molecule RALBP1 mediates the effect of the mitotic cyclin, Cyclin B1, on Drp1 [26, 27]. Although, Ras-ERK signaling driven regulation of Drp1 contributes to cell transformation [25, 45], any relationship to cell cycle alteration has not been reported. Our exploratory analyses of the publically available gene expression data from the 31 cancer types in TCGA reveal that Drp1 is predominantly co-expressed with genes involved in cell cycle along with those involved in gene expression and metabolism, across majority of the cancer types (Figure 1). We further focused on epithelial ovarian cancer (EOC) given observations that FoxM1 driven cell cycle pathway is perturbed in these very patients from TCGA [34] and our previous observation on an active role of Drp1 in ovarian epithelial cell layer development in *Drosophila* [23]. More detailed stringent analyses confirmed the co-expression of Drp1 specifically with genes involved in mitotic transition of cell cycle in the TCGA-EOC tumors (Figure 3), including the mitotic transcription factor, FoxM1 (Supplementary Figure 3). Since, Drp1 co-expression module constitutes of genes that can be upstream (or downstream) of Drp1′s action, we speculate that FoxM1 can potentially regulate Drp1 gene expression in a cell cycle dependent manner, at least in EOC cells. We also find that Mff, which recruits Drp1 to the mitochondrial membrane towards causing mitochondrial fission [46], overwhelmingly shares its cell cycle co-expression module with Drp1. Moreover, none of the mitochondrial fusion proteins examined exhibit any negative correlation with cell cycle molecules. Therefore, our results raise the possibility that the cell cycle regulation, at least in EOC tissues, occurs primarily at the level of Drp1 mediated mitochondrial fission promoted by mitochondrial recruitment of Drp1 by Mff, which remains to be tested. Consistently, our laboratory experimentations involving repression of Drp1 establishes a potential causal role of Drp1 in mitotic transition and cell proliferation in EOC cells (Figure 3, Supplementary Figure 7). Previous studies from our laboratory and others demonstrated that Drp1 regulates the levels of the cell cycle molecule Cyclin E [24–28, 36], which has been identified as a driver gene in the TCGA ovarian cancer cohort [34]. Since Drp1 is already emerging as a chemotherapeutic target [47], our current findings strongly encourage detailed investigation of the mechanisms underlying the crosstalk between Drp1 and cell cycle in various tumor types.

The significance of the Drp1-Cell Cycle co-expressing module becomes more evident since we detected it in the chemosensitive but not in the relatively chemoresistant primary EOC tumors, irrespective of Drp1 expression levels (Figure 4). The potential Drp1 driven boost in mitosis in the primary tumors may underlie their ability to respond to the platinum-taxane based chemotherapy that majorly targets the proliferating cells [40], while lack of the potential Drp1 driven boost in mitosis may underlie the lack of robust response of the primary tumors. It is possible that the involvement of Drp1 is different in the resistant EOC patients, as indicated by the predominance of genes involved in signal transduction in the Drp1-coexpression module in this group (Figure 4E). This dichotomy in Drp1′s role is also evident in our finding that Drp1 is associated with cell cycle boost in some specific relapsed resistant patients (Drp1-High), but not in others (Drp1-Low) (Figure 5). Further investigation is needed to understand the mechanisms underlying how the Drp1′s involvement in mitosis, or lack of it thereof, can be related to primary or acquired chemoresistance. It is noteworthy in this context that Drp1 repression can attenuate induction of mitochondria dependent apoptosis [18, 48], which can potentially cause chemoresistance [40, 49], although such a role of Drp1 may not be universal [8]. From our results we speculate that Drp1 may exert a pro-apoptotic role in Drp1-Low and an anti-apoptotic role in Drp1-High patients. We propose that the Drp1-based-gene-expression-signature, identified by the comparison of the Drp1-High and Drp1-Low patients (Supplementary Figure 13A), is the first step towards elucidation of the potential influence of the genetic background in guiding Drp1′s role as pro or anti-apoptotic.

Our data obtained from comparison of the primary and relapsed ICGC samples are end point analyses and therefore preclude us from concluding about any role of Drp1 in chemoresistance acquisition. Drp1′s involvement is emerging to be important in stem cell regulation [30, 50–51, 36] and cancer stem cells (CSCs) have been shown to contribute to acquisition of chemoresistance [40]. Our comparison of expression of a functional marker for ovarian CSCs, Aldh1A1, between primary and relapse samples demonstrated an inverse relationship between Aldh1A1 and Drp1 expression (Supplementary Figure 9B). This finding is consistent with our previous observation that transient repression of Drp1 in ovarian cancer cells leads to elevation of Aldh1A levels in ovarian cancer cells [36]. Therefore, our current data from EOC patients raises an exciting possibility that modulation of Drp1 may alter the Aldh1A1 levels to potentially modulate stem cell properties at least in the relapsed EOC disease. However, we did not detect any difference of a potential CSC gene expression signature (n=68) [52–54] in the primary tissue of the sensitive, recurred-resistant and resistant patients (Supplementary Figure 15A), which could be masked by the overwhelming presence of the bulk tumor cells over the limited number of CSCs.

Ovarian cancer is associated with marked levels of inter patient heterogeneity although greater than 80% of the deaths are due to relapse with chemoresistant tumors [33]. Therefore, focus in ovarian cancer research has shifted towards testing new therapeutic strategies in a more precise and personalized manner [55, 56]. In this light our identification of TCGA-RR-70 and TCGA-RR-48 group of patients, by applying a Drp1-based-gene-expression signature, is highly significant (Figure 6). Importantly, our data implies that 60% of the recurred-resistant population (TCGA-RR-70) show poor survival outcome than that of the rest (TCGA-RR-48) due to a potential activation of Drp1 driven cell cycle in their relapsed tumors. We propose that a Drp1-based-gene-expression-signature, in its current or more refined form, can potentially identify EOC patients from their primary tumors who may have better or worse survival after exposure to a platinum-taxane based chemotherapy. Such knowledge from the primary tumors may be used to design better chemotherapeutic or targeted strategies to overcome chemoresistance.

Use of mitochondrial inhibitors to prevent occurrence of chemoresistance has been recently demonstrated in pancreatic cancer model systems [57, 58]. Our current findings on the mitochondrial regulation of cell cycle through Drp1 highlights an important role of mitochondria in ovarian cancer chemoresistance and relapse. We find that certain EOC patients (Drp1-High) that have potentially elevated mitochondrial energetics in their primary tumors undergo activation of Drp1 related Cell Cycle regime in their relapsed disease (Figure 5), which can potentially lead to their poor survival after chemotherapy (Figure 6). Chemotherapeutic drugs have been previously shown to affect the function of Drp1 [49]. Along those lines we noted that exposure to platinum based chemotherapeutic drug can enhance the inhibitory phosphorylation of Drp1 in an ovarian cancer cell line (Supplementary Figure 15b). Our data also raise the possibility that chemotherapy may have different effects on Drp1 in the Drp1-High and Drp1-Low patients. We speculate that a Drp1-based-gene-expression signature may be able to identify EOC patients that would specifically benefit by replacement of platinum-taxane based chemotherapeutics with a mitochondria based targeted therapy.

Overall, our study indicates that analyses of Drp1 mRNA expression can reveal functional aspects of the molecule and also demonstrate the potential usefulness of such analyses in translational endeavors involving Drp1. In summary, our Drp1 based analyses of the publically available cancer genomes highlights that Drp1 driven cell cycle regulation is a general feature of various cancer types, which may allow robust response to chemotherapeutics targeted against proliferating cells. Based on our current data we speculate that preventing further activation of Drp1 driven mitosis may be critical in preventing relapse in a group of patients that can be identified by a Drp1-based-gene-expression-signature employed on their primary tumors.

## MATERIALS AND METHODS

### Data types and sources

TCGA RNA-seqV2 data was downloaded from TCGA data matrix (https://tcga-data.nci.nih.gov/tcga/dataAccessMatrix.htm). Raw FASTQ files were downloaded from CGHUB (https://cghub.ucsc.edu) and reanalyzed using in-house pipeline. ICGC RNA-Seq data was downloaded from the European Genome-phenome Archive (EGA; https://www.ebi.ac.uk/ega/home; accession EGAD00001000877). GISTIC data was downloaded from Broad Institute's firehose pipeline using firehose_get script (https://confluence.broadinstitute.org/display/GDAC/Download).

TCGA EOC patients were clinically classified based on their reported “ProgressFreeStatus” and “PlatinumStatus” [34]. 33 cases labelled “DiseaseFree” for “ProgressionFreeStatus” and “sensitive” for “PlatinumStatus” were deemed “Sensitive”; 70 cases labelled “resistant” for “PlatinumStatus” were deemed “Resistant”; 223 cases that were “sensitve” for “PlatinumStatus” category and labelled as “Recurred/Progressed” for “ProgressionFreeStatus” were deemed “Recurred-Resistant”. Thus, the degree of patient chemotherapeutic response is as follows: Sensitive>Recurred-Resistant>Resistant. Patient survival data was obtained from the TCGA data matrix.

### RNA-seq data processing and expression analyses

Paired end reads in FASTQ files were aligned to the human reference genome (hg19) using STAR aligner (version 2.4.2a) [59]. The resulting BAM files were then used to generate the gene and transcript level expression estimate using RSEM (version 1.2.21) [60]. The “estimated count” data was normalized using upper-quartile normalization, re-scaled and log2 converted. The BAM files provided by EGA were sorted by read name using Samtools (version 1.3) [61] and then paired-ends reads were extracted using Bedtools (version 2.23) [62] to estimate expression as described above. Outlier correction (MAD) was applied on the 430 TCGA-EOC samples where 215 samples resided within within +/− 1 MAD for Drp1 expression. Only 175 Drp1-MAD1 patients had clinical outcome and thus included in our analyses. Statistical significance of the difference of the median Drp1 expression amongst categories was determined using pairwise permutation test by randomly shuffling the category labels.

### GSEA

GSEA was performed using the Broad institute software (http://software.broadinstitute.org/gsea/index.jsp). A “pre-ranked” list of genes based on either gene correlation or differential expression was provided for enrichment analysis using REACTOME and KEGG pathway databases from the MSigDB (Molecular Signatures Database) [63]. The GSEA run parameters are: permutations-1000, collapse data to gene symbol-false, enrichment statistics-classic, max-size-500 and min-size-15.

Given that the REACTOME pathway is not perfect we considered the following re-assignments of genes: a) the REACTOME top-level DNA Replication under REACTOME Cell Cycle since DNA replication is a core cell cycle functionality; b) two new top-level categories called “Ribosomes” and “Histones” to reassign all the various pathways that were identified primarily based on the ribosomal and histone genes, respectively. We re-assigned genes belonging to multiple REACTOME pathways to a unique pathway as follows: we first calculated average correlation (see below) of the gene with all other genes within each conflicting category, then the gene was assigned to the category that had the maximum average correlation value for the gene.

### Gene correlation and differential gene expression analyses

We performed Pearson correlation of DNM1L with all other genes using log2 converted normalized expression value. For representing gene by gene correlation matrix using heatmap, the matrix were converted to a distance matrix using the following formula: D(i,j)=1− (Cor(i,j)+1)/2, where, *D(i, j)* is the distance and *Cor(i,j)* is the correlation between genes *i, j*. The formula effectively maps the correlation values (1 and -1) between distance (1 and 0). This distance matrix was then directly used to draw the heatmap using R package “pheatmap”.

We performed differential gene expression analysis of the upper quartile normalized gene expression data using edgeR Bioconductor package [64] and log fold change values were used for ranking genes for GSEA.

### Non Negative Matrix Factorization (NMF)

NMF is a factorization algorithm that splits a given matrix into two matrices [65]. Here, we applied NMF to split a gene by sample matrix (*m* × *n*) into a gene by rank (*m* × *k*) and a rank by sample (*k* × *n*) matrices. The NMF was performed using NMF package in R [65] and consensus matrix was obtained with 100 runs. The optimal rank (*k*) was estimated by evaluating various output parameters, most importantly the cophenetic and the silhouette scores between the NMF runs with varying rank (*k* = 2 to 10).

### Co-clustering of ICGC and TCGA samples

We used “pvclust” [66] package from R to cluster the samples using “ward.D2” option using the 745 gene expression signature. For testing the robustness of the cluster, we did bootstrap analysis with 1000 replicates. The final circular tree was drawn first generating a newick tree file from the pvclust object and then using the FigTree software (http://tree.bio.ed.ac.uk/software/figtree/).

### Patient survival analyses

Survival probabilities were determined using R package “survival”. Survival information (time to death) was available for 21 patients from the TCGA-RR-70 and 23 patients from TCGA-RR-48. We used “days to death” values for calculating survival probabilities after diagnoses. We used the interval between the “earliest chemotherapy start” and the “days to death” value for each patient to obtain the survival probabilities after chemotherapy commencement. Statistical significance was obtained using chi square test.

### Cell culture and related procedures

A2780 ovarian cancer cells were cultured in RPMI medium using standard laboratory methods.

For knocking down Drp1 expression cells were lentivirally transduced with Drp1 or NT shRNA [36] and stable lines were selected using puromycin. Flow cytometry based cell cycle analyses were performed as described before [36]. For colony assay, 500 cells were seeded in each well of 6 well plates and colony growth quantified by crystal violet staining as before [36]. Cell proliferation rate was obtained by normalization the crystal violet counts of 10 day cell growth by that of 7 days. For transient knockdown experiments transfection of plasmids or transduction of lentiviral particles encoding non-targeting or Drp1 targeted shRNAs were performed for 72 hours. Hereafter, cells were harvested for flow cytometry or re-plated for cell proliferation assay by seeding 50,000 A2780 and 30,000 SKOV3 cells in each well of the 24 well plates and harvesting them for crystal violet staining after 24 and 72 hours post seeding.

### Statistical analyses

All statistical analyses were performed using R statistical packages (v3.2.2).

## SUPPLEMENATRY FIGURES AND TABLES























## References

[1] Hanahan D, Weinberg RA (2011). Hallmarks of cancer: the next generation. Cell.

[2] Gottlieb E, Tomlinson IP (2005). Mitochondrial tumour suppressors: a genetic and biochemical update. Nat Rev Cancer.

[3] Weinberg SE, Chandel NS (2014). Targeting mitochondria metabolism for cancer therapy. Nat Chem Biol.

[4] Nunnari J, Suomalainen A (2012). Mitochondria: in sickness and in health. Cell.

[5] Mitra K (2013). Mitochondrial fission-fusion as an emerging key regulator of cell proliferation and differentiation. Bioessays.

[6] Hoppins S The regulation of mitochondrial dynamics. Curr Opin Cell Biol.

[7] Senft D, Ronai ZA (2016). Regulators of mitochondrial dynamics in cancer. Curr Opin Cell Biol.

[8] Kageyama Y, Zhang Z, Sesaki H (2011). Mitochondrial division: molecular machinery and physiological functions. Curr Opin Cell Biol.

[9] Palmer CS, Osellame LD, Laine D, Koutsopoulos OS, Frazier AE, Ryan MT (2011). MiD49 and MiD51 new components of the mitochondrial fission machinery. EMBO Rep.

[10] Martin OJ, Lai L, Soundarapandian MM, Leone TC, Zorzano A, Keller MP, Attie AD, Muoio DM, Kelly DP (2013). A role for peroxisome proliferator-activated receptor gamma coactivator-1 in the control of mitochondrial dynamics during postnatal cardiac growth. Circ Res.

[11] Choudhary V, Kaddour-Djebbar I, Lakshmikanthan V, Ghazaly T, Thangjam GS, Sreekumar A, Lewis RW, Mills IG, Bollag WB, Kumar MV (2011). Novel role of androgens in mitochondrial fission and apoptosis. Mol Cancer Res.

[12] Mai S, Klinkenberg M, Auburger G, Bereiter-Hahn J, Jendrach M (2010). Decreased expression of Drp1 and Fis1 mediates mitochondrial elongation in senescent cells and enhances resistance to oxidative stress through PINK1. J Cell Sci.

[13] Wan YY, Zhang JF, Yang ZJ, Jiang LP, Wei YF, Lai QN, Wang JB, Xin HB, Han XJ (2014). Involvement of Drp1 in hypoxia-induced migration of human glioblastoma U251 cells. Oncol Rep.

[14] Richter V, Singh AP, Kvansakul M, Ryan MT, Osellame LD (2015). Splitting up the powerhouse: structural insights into the mechanism of mitochondrial fission. Cell Mol Life Sci.

[15] Kageyama Y, Zhang Z, Roda R, Fukaya M, Wakabayashi J, Wakabayashi N, Kensler TW, Reddy PH, Iijima M, Sesaki H (2012). Mitochondrial division ensures the survival of postmitotic neurons by suppressing oxidative damage. J Cell Biol.

[16] Song M, Dorn GW (2015). Mitoconfusion: noncanonical functioning of dynamism factors in static mitochondria of the heart. Cell Metab.

[17] Udagawa O, Ishihara T, Maeda M, Matsunaga Y, Tsukamoto S, Kawano N, Miyado K, Shitara H, Yokota S, Nomura M, Mihara K, Mizushima N, Ishihara N (2014). Mitochondrial fission factor Drp1 maintains oocyte quality via dynamic rearrangement of multiple organelles. Curr Biol.

[18] Hoppins S, Nunnari J (2012). Cell Biology Mitochondrial dynamics and apoptosis--the ER connection. Science.

[19] Twig G, Elorza A, Molina AJ, Mohamed H, Wikstrom JD, Walzer G, Stiles L, Haigh SE, Katz S, Las G, Alroy J, Wu M, Py BF, Yuan J, Deeney JT, Corkey BE (2008). Fission and selective fusion govern mitochondrial segregation and elimination by autophagy. EMBO J.

[20] Liesa M, Shirihai OS (2013). Mitochondrial dynamics in the regulation of nutrient utilization and energy expenditure. Cell Metab.

[21] Wang X, Jiang W, Yan Y, Gong T, Han J, Tian Z, Zhou R (2014). RNA viruses promote activation of the NLRP3 inflammasome through a RIP1-RIP3-DRP1 signaling pathway. Nat Immunol.

[22] Kang YJ, Bang BR, Han KH, Hong L, Shim EJ, Ma J, Lerner RA, Otsuka M (2015). Regulation of NKT cell-mediated immune responses to tumours and liver inflammation by mitochondrial PGAM5-Drp1 signalling. Nat Commun.

[23] Mitra K, Rikhy R, Lilly M, Lippincott-Schwartz J (2012). DRP1-dependent mitochondrial fission initiates follicle cell differentiation during Drosophila oogenesis. J Cell Biol.

[24] Mitra K, Wunder C, Roysam B, Lin G, Lippincott-Schwartz J (2009). A hyperfused mitochondrial state achieved at G1-S regulates cyclin E buildup and entry into S phase. Proc Natl Acad Sci U S A.

[25] Serasinghe MN, Wieder SY, Renault TT, Elkholi R, Asciolla JJ, Yao JL, Jabado O, Hoehn K, Kageyama Y, Sesaki H, Chipuk JE (2015). Mitochondrial Division Is Requisite to RAS-Induced Transformation and Targeted by Oncogenic MAPK Pathway Inhibitors. Mol Cell.

[26] Taguchi N, Ishihara N, Jofuku A, Oka T, Mihara K (2007). Mitotic phosphorylation of dynamin-related GTPase Drp1 participates in mitochondrial fission. J Biol Chem.

[27] Kashatus DF, Lim KH, Brady DC, Pershing NL, Cox AD, Counter CM (2011). RALA and RALBP1 regulate mitochondrial fission at mitosis. Nat Cell Biol.

[28] Qian W, Choi S, Gibson GA, Watkins SC, Bakkenist CJ, Houten BV (2012). Mitochondrial hyperfusion induced by loss of fission protein Drp1 causes ATM-dependent G2/M arrest and aneuploidy through DNA replication stress. J Cell Sci.

[29] Jirawatnotai S, Hu Y, Michowski W, Elias JE, Becks L, Bienvenu F, Zagozdzon A, Goswami T, Wang YE, Clark AB, Kunkel TA, van Harn T, Xia B, Correll M, Quackenbush J, Livingston DM (2011). A function for cyclin D1 in DNA repair uncovered by protein interactome analyses in human cancers. Nature.

[30] Xie Q, Wu Q, Horbinski CM, Flavahan WA, Yang K, Zhou W, Dombrowski SM, Huang Z, Fang X, Shi Y, Ferguson AN, Kashatus DF, Bao S, Rich JN (2015). Mitochondrial control by DRP1 in brain tumor initiating cells. Nat Neurosci.

[31] Zhao J, Zhang J, Yu M, Xie Y, Huang Y, Wolff DW, Abel PW, Tu Y (2012). Mitochondrial dynamics regulates migration and invasion of breast cancer cells. Oncogene.

[32] Rehman J, Zhang HJ, Toth PT, Zhang Y, Marsboom G, Hong Z, Salgia R, Husain AN, Wietholt C, Archer SL (2012). Inhibition of mitochondrial fission prevents cell cycle progression in lung cancer. FASEB J.

[33] Ozols RF, Bookman MA, Connolly DC, Daly MB, Godwin AK, Schilder RJ, Xu X, Hamilton TC (2004). Focus on epithelial ovarian cancer. Cancer Cell.

[34] (2011). TCGA Integrated genomic analyses of ovarian carcinoma. Nature.

[35] Mermel CH, Schumacher SE, Hill B, Meyerson ML, Beroukhim R, Getz G (2011). GISTIC2 0 facilitates sensitive and confident localization of the targets of focal somatic copy-number alteration in human cancers. Genome Biol.

[36] Parker DJ, Iyer A, Shah S, Moran A, Hjelmeland AB, Basu MK, Liu R, Mitra K (2015). A new mitochondrial pool of cyclin E regulated by Drp1 is linked to cell-density-dependent cell proliferation. J Cell Sci.

[37] Bosquet JG, Marchion DC, Chon H, Lancaster JM, Chanock S (2014). Analysis of chemotherapeutic response in ovarian cancers using publicly available high-throughput data. Cancer Res.

[38] Westrate LM, Sayfie AD, Burgenske DM, MacKeigan JP (2014). Persistent mitochondrial hyperfusion promotes G2/M accumulation and caspase-dependent cell death. PLoS One.

[39] Patch AM, Christie EL, Etemadmoghadam D, Garsed DW, George J, Fereday S, Nones K, Cowin P, Alsop K, Bailey PJ, Kassahn KS, Newell F, Quinn MC, Kazakoff S, Quek K, Wilhelm-Benartzi C (2015). Whole-genome characterization of chemoresistant ovarian cancer. Nature.

[40] Holohan C, Van Schaeybroeck S, Longley DB, Johnston PG (2013). Cancer drug resistance: an evolving paradigm. Nat Rev Cancer.

[41] Flesken-Nikitin A, Hwang CI, Cheng CY, Michurina TV, Enikolopov G, Nikitin AY (2013). Ovarian surface epithelium at the junction area contains a cancer-prone stem cell niche. Nature.

[42] Landen CN, Goodman B, Katre AA, Steg AD, Nick AM, Stone RL, Miller LD, Mejia PV, Jennings NB, Gershenson DM, Bast RC, Coleman RL, Lopez-Berestein G, Sood AK (2010). Targeting aldehyde dehydrogenase cancer stem cells in ovarian cancer. Mol Cancer Ther.

[43] Merrill RA, Dagda RK, Dickey AS, Cribbs JT, Green SH, Usachev YM, Strack S (2011). Mechanism of neuroprotective mitochondrial remodeling by PKA/AKAP1. PLoS Biol.

[44] Toyama EQ, Herzig S, Courchet J, Lewis TL, Loson OC, Hellberg K, Young NP, Chen H, Polleux F, Chan DC, Shaw RJ (2016). Metabolism AMP-activated protein kinase mediates mitochondrial fission in response to energy stress. Science.

[45] Kashatus JA, Nascimento A, Myers LJ, Sher A, Byrne FL, Hoehn KL, Counter CM, Kashatus DF (2015). Erk2 Phosphorylation of Drp1 Promotes Mitochondrial Fission and MAPK-Driven Tumor Growth. Mol Cell.

[46] Otera H, Wang C, Cleland MM, Setoguchi K, Yokota S, Youle RJ, Mihara K (2010). Mff is an essential factor for mitochondrial recruitment of Drp1 during mitochondrial fission in mammalian cells. J Cell Biol.

[47] Qian W, Wang J, Van Houten B (2013). The role of dynamin-related protein 1 in cancer growth: a promising therapeutic target?. Expert Opin Ther Targets.

[48] Youle RJ, van der Bliek AM (2012). Mitochondrial fission fusion and stress. Science.

[49] Wang JX, Li Q, Li PF (2009). Apoptosis repressor with caspase recruitment domain contributes to chemotherapy resistance by abolishing mitochondrial fission mediated by dynamin-related protein-1. Cancer Res.

[50] Todd LR, Damin MN, Gomathinayagam R, Horn SR, Means AR, Sankar U (2010). Growth factor erv1-like modulates Drp1 to preserve mitochondrial dynamics and function in mouse embryonic stem cells. Mol Biol Cell.

[51] Prieto J, Leon M, Ponsoda X, Sendra R, Bort R, Ferrer-Lorente R, Raya A, Lopez-Garcia C, Torres J (2016). Early ERK1/2 activation promotes DRP1-dependent mitochondrial fission necessary for cell reprogramming. Nat Commun.

[52] Ng A, Barker N (2015). Ovary and fimbrial stem cells: biology niche and cancer origins. Nat Rev Mol Cell Biol.

[53] Ishiguro T, Sato A, Ohata H, Ikarashi Y, Takahashi R, Ochiya T, Yoshida M, Tsuda H, Onda T, Kato T, Kasamatsu T, Enomoto T, Tanaka K, Nakagama H, Okamoto K (2015). Establishment and Characterization of an In Vitro Model of Ovarian Cancer Stem-like Cells with an Enhanced Proliferative Capacity. Cancer Res.

[54] Pece S, Tosoni D, Confalonieri S, Mazzarol G, Vecchi M, Ronzoni S, Bernard L, Viale G, Pelicci PG, Di Fiore PP (2010). Biological and Molecular Heterogeneity of Breast Cancers Correlates with Their Cancer Stem Cell Content. Cell.

[55] Bashashati A, Ha G, Tone A, Ding J, Prentice LM, Roth A, Rosner J, Shumansky K, Kalloger S, Senz J, Yang W, McConechy M, Melnyk N, Anglesio M, Luk MT, Tse K (2013). Distinct evolutionary trajectories of primary high-grade serous ovarian cancers revealed through spatial mutational profiling. J Pathol.

[56] Banerjee S, Kaye SB (2013). New strategies in the treatment of ovarian cancer: current clinical perspectives and future potential. Clin Cancer Res.

[57] Sancho P, Burgos-Ramos E, Tavera A, Bou Kheir T, Jagust P, Schoenhals M, Barneda D, Sellers K, Campos-Olivas R, Grana O, Viera CR, Yuneva M, Sainz B, Heeschen C (2015). MYC/PGC-1alpha Balance Determines the Metabolic Phenotype and Plasticity of Pancreatic Cancer Stem Cells. Cell Metab.

[58] Viale A, Pettazzoni P, Lyssiotis CA, Ying H, Sanchez N, Marchesini M, Carugo A, Green T, Seth S, Giuliani V, Kost-Alimova M, Muller F, Colla S, Nezi L, Genovese G, Deem AK (2014). Oncogene ablation-resistant pancreatic cancer cells depend on mitochondrial function. Nature.

[59] Dobin A, Davis CA, Schlesinger F, Drenkow J, Zaleski C, Jha S, Batut P, Chaisson M, Gingeras TR (2012). STAR: ultrafast universal RNA-seq aligner. Bioinformatics.

[60] Li B, Dewey CN (2011). RSEM: accurate transcript quantification from RNA-Seq data with or without a reference genome. BMC Bioinformatics.

[61] Li H, Handsaker B, Wysoker A, Fennell T, Ruan J, Homer N, Marth G, Abecasis G, Durbin R (2009). The Sequence Alignment/Map format and SAMtools. Bioinformatics.

[62] Quinlan AR, Hall IM (2010). BEDTools: a flexible suite of utilities for comparing genomic features. Bioinformatics.

[63] Subramanian A, Tamayo P, Mootha VK, Mukherjee S, Ebert BL, Gillette MA, Paulovich A, Pomeroy SL, Golub TR, Lander ES, Mesirov JP (2005). Gene set enrichment analysis: a knowledge-based approach for interpreting genome-wide expression profiles. Proc Natl Acad Sci U S A.

[64] Gentleman RC, Carey VJ, Bates DM, Bolstad B, Dettling M, Dudoit S, Ellis B, Gautier L, Ge Y, Gentry J, Hornik K, Hothorn T, Huber W, Iacus S, Irizarry R, Leisch F (2004). Bioconductor: open software development for computational biology and bioinformatics. Genome Biol.

[65] Gaujoux R, Seoighe C (2010). A flexible R package for nonnegative matrix factorization. BMC Bioinformatics.

[66] Suzuki R, Shimodaira H (2006). Pvclust: an R package for assessing the uncertainty in hierarchical clustering. Bioinformatics.

