# Supplementary material for: Crosstalk between the mitochondrial fission protein, Drp1, and the cell cycle is identified across various cancer types and can impact survival of epithelial ovarian cancer patients
Source: Oncotarget. 2016 Aug 4;7(37):60021–37. doi: 10.18632/oncotarget.11047 (PMC5312366; doi:10.18632/oncotarget.11047)
Supplement: Supplementary file 1 [file oncotarget-07-60021-s001.pdf]

Crosstalk between the mitochondrial fission protein, Drp1, and the cell cycle is identified across various cancer types and can impact survival of epithelial ovarian cancer patients

SUPPLEMENATRY FIGURES AND TABLES

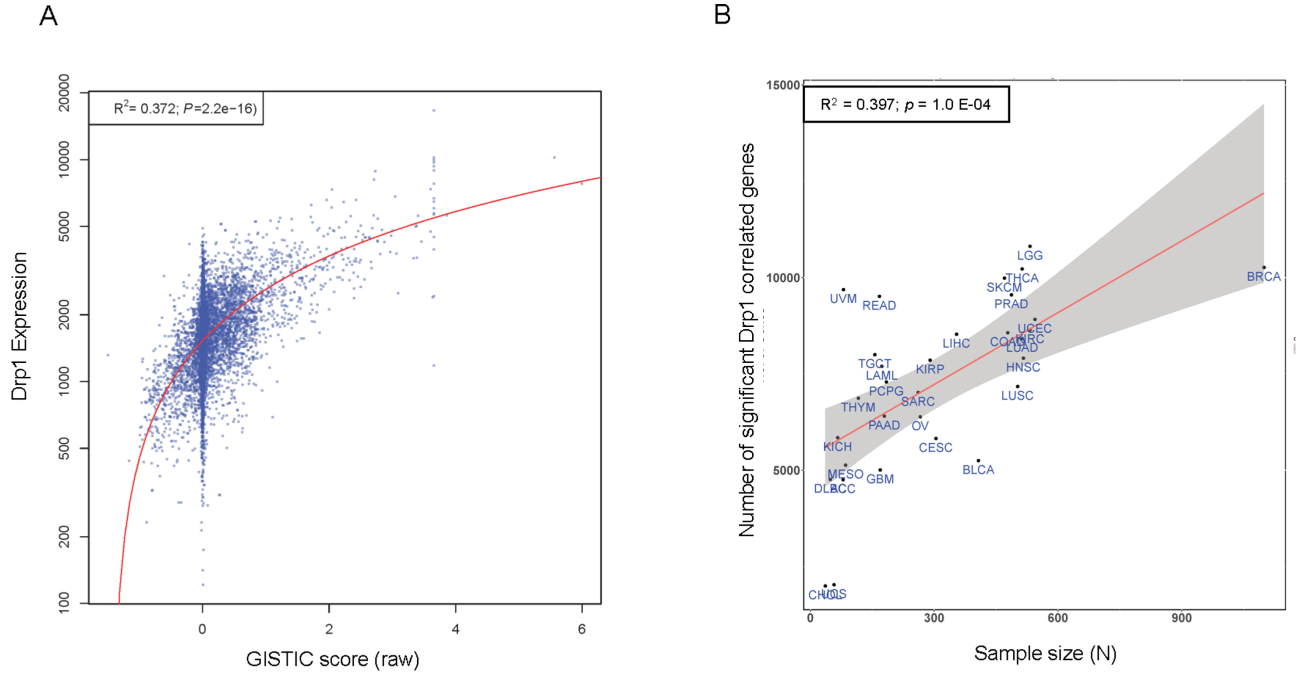

Supplementary Figure S1: A. Linear regression analysis of Drp1 expression (plotted on a log scale) and raw GISTIC scores in individual patients in various TCGA cohorts (blue dots). **B.** Linear regression analysis of the patient sample size of the TCGA cohorts and the number of statistically significant Drp1 correlated genes in each cancer cohort (blue dots). Grey area signifies 95% confidence interval. Red lines represent the regression line with the Regression coefficient (R^2), p value mentioned on top left.

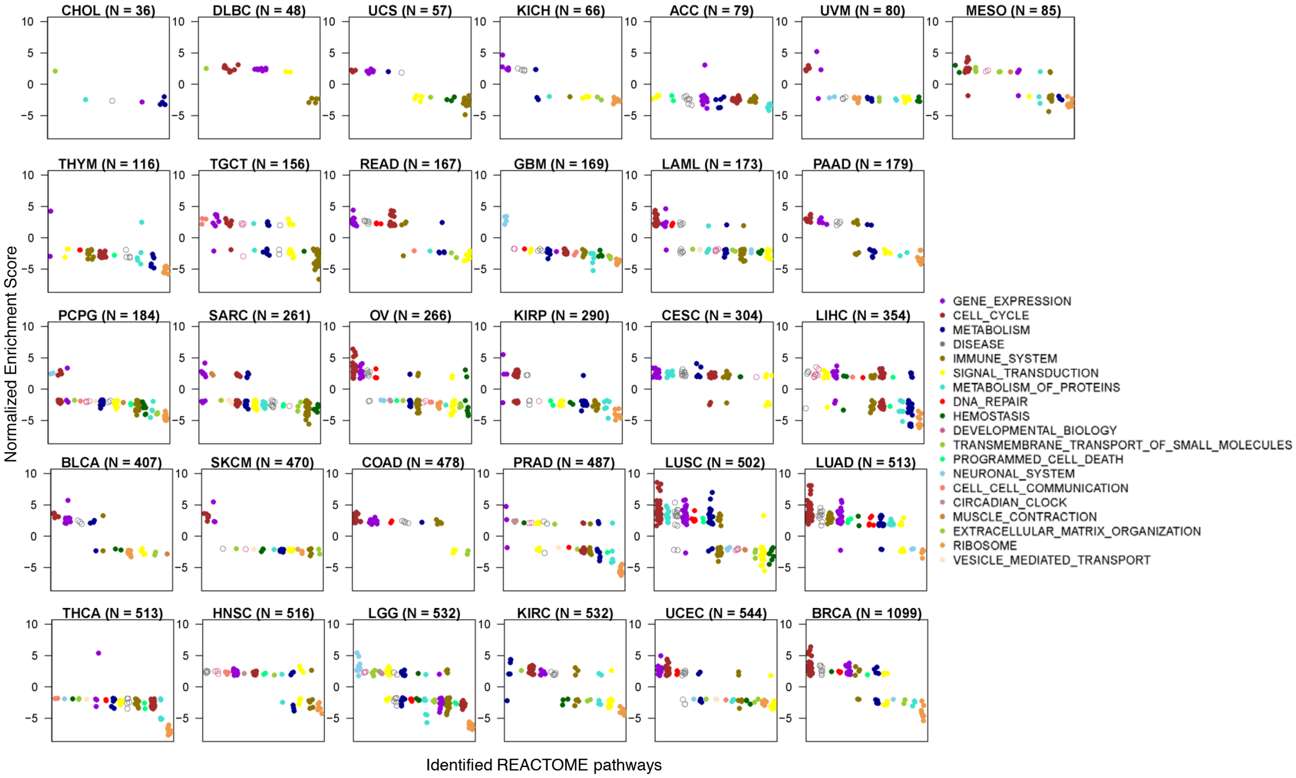

Supplementary Figure S2: Dot plots of the normalized enrichment scores (Y axes) for the identified Drp1 correlating REACTOME pathways (X axes) in individual TCGA cancer types. Positive and negative NES scores signify positive and negative correlation, respectively, with Drp1. The color coding represents the top-level category for each of the identified pathway in the index. N depicts sample size of each cancer type.

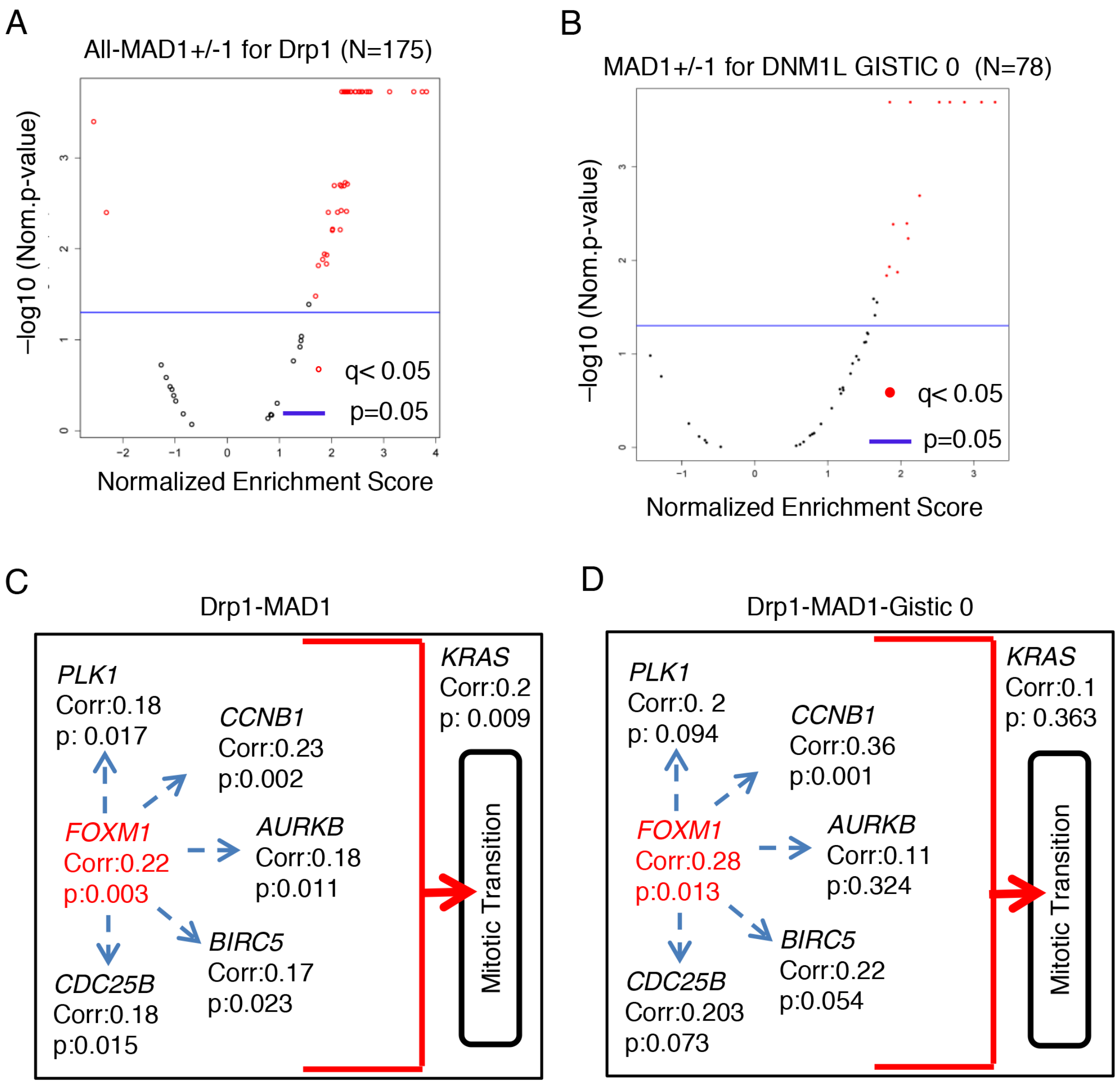

Supplementary Figure S3: A. Volcano plot representing the output of the GSEA analyses of statistically significant Drp1 correlated genes in the TCGA EOC patients. Negatively and positively correlated pathways have negative and positive Normalized Enrichment Scores respectively. N depicts number of patients. **B.** Volcano plot representing the output of the GSEA analyses of statistically significant Drp1 correlated genes in the TCGA EOC patients with 0 GISTIC scores. Negatively and positively correlated pathways have negative and positive Normalized Enrichment Scores respectively. N depicts number of patients. **C.** Drp1 correlation (corr) with FoxM1 transcription factors and its key downstream targets regulating mitotic transition of the cell cycle in the Drp1-MAD1 patient group. Correlation of Drp1 with KRAS is also shown, although it is not part of the FoxM1 pathway. **D.** Drp1 correlation (corr) with FoxM1 transcription factors and its key downstream targets regulating mitotic transition of the cell cycle in the Drp1-MAD1-GISTIC 0 patient group. Correlation of Drp1 with KRAS is also shown, although it is not part of the FoxM1 pathway.

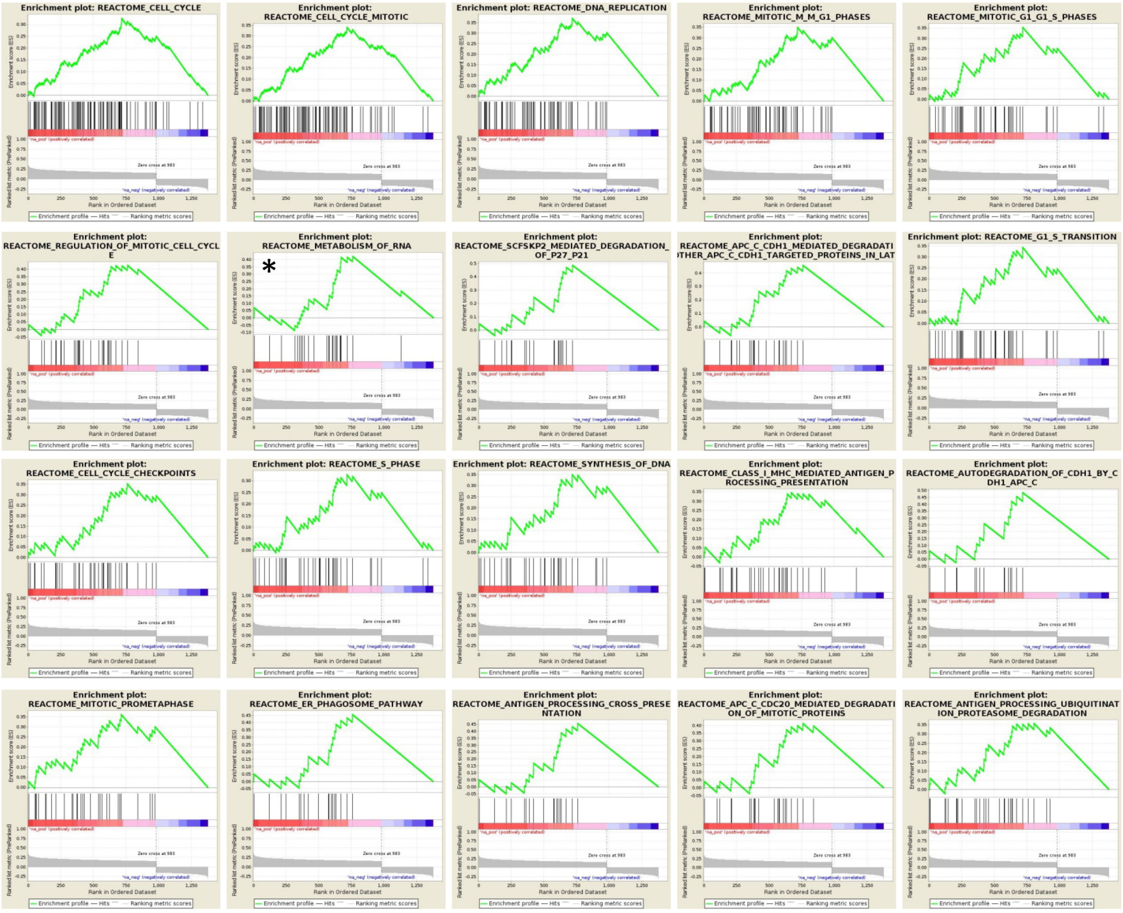

Supplementary Figure S4: GSEA enrichment plots of the top 20 Reactome pathways identified using genes and their correlation values with Drp1 expression in the TCGA EOC patients. * signifies reassignment to the REACTOME "Metabolism of Proteins" category since the gene list included predominantly proteasomal components of that pathway.

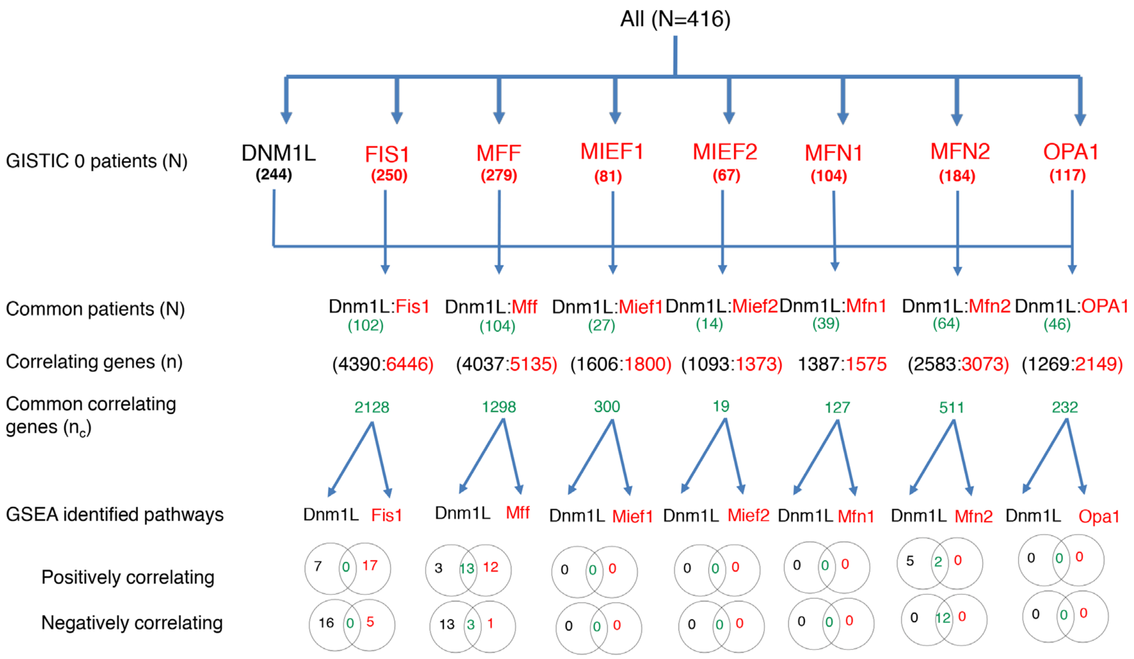

Supplementary Figure S5: Analyses scheme for comparing genome wide correlation profile of Drp1 and other mitochondrial fission and fusion proteins. The various level of pairwise comparison are mentioned on the left with the corresponding sample numbers (N) or gene numbers (n) for each comparison on the right. The numbers in the Venn diagram represent the total number of REACTOME pathways identified using Dnm1L correlation values of the genes coomon with the other gene of interest and that using the correlation values of that gene.

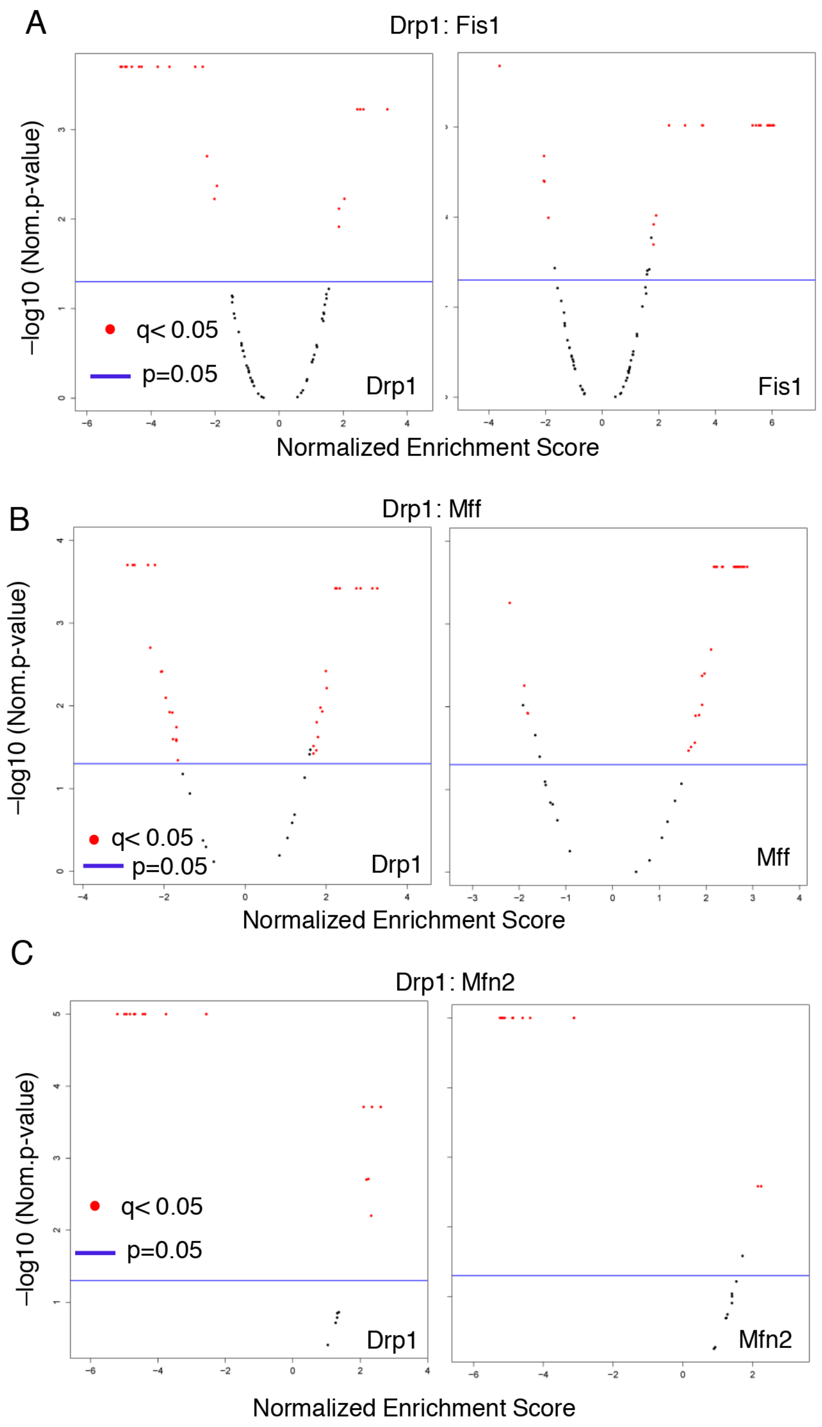

Supplementary Figure S6: A. Volcano plot representing the output of the GSEA analyses of statistically significant Drp1 and Fis1 commonly correlated genes in the TCGA EOC patients. **B.** Volcano plot representing the output of the GSEA analyses of statistically significant Drp1 and Mff commonly correlated genes in the TCGA EOC patients. **C.** Volcano plot representing the output of the GSEA analyses of statistically significant Drp1 and Mfn2 commonly correlated genes in the TCGA EOC patients. Negatively and positively correlated pathways have negative and positive Normalized Enrichment Scores respectively.

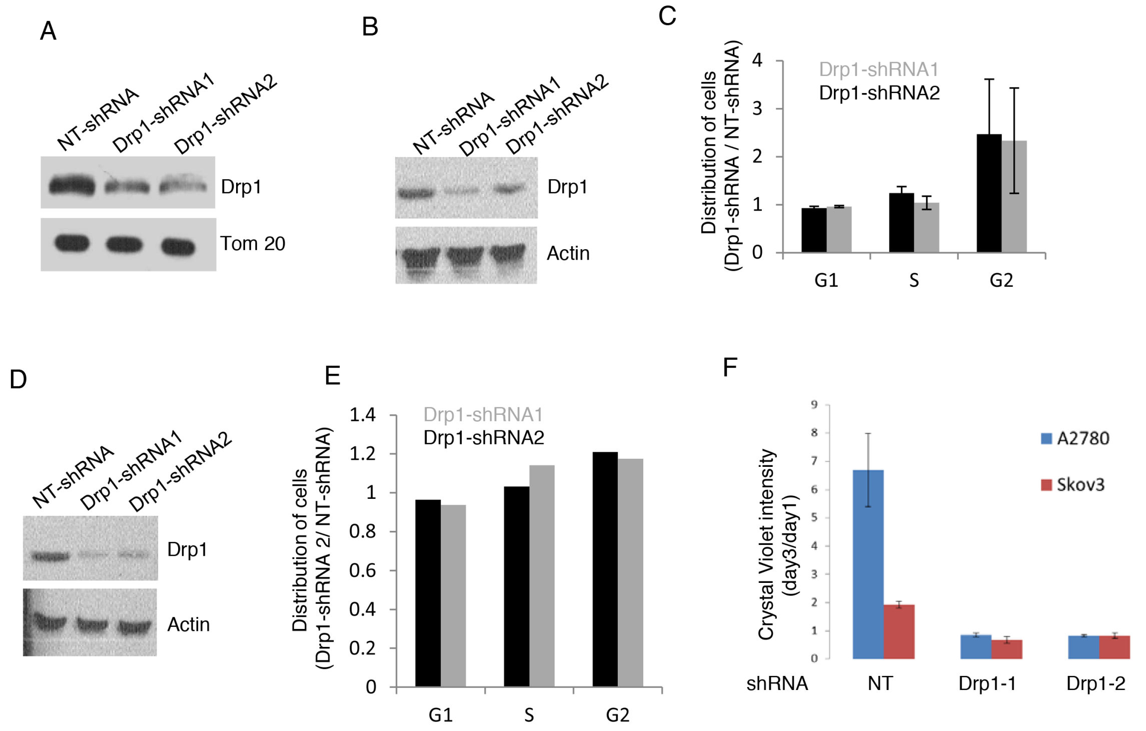

Supplementary Figure S7: A. Immunoblot showing stable knockdown of Drp1 protein levels using two distinct Drp1 shRNAs as compared to that of a non-targeting (NT) shRNA in A2780 cell line. Tom20 is used as a loading control. **B.** Immunoblot showing plasmid mediated transient knockdown of Drp1 protein levels using two distinct Drp1 shRNAs as compared to that of a non-targeting (NT) shRNA in A2780 cell line. Actin is used as a loading control. **C.** Bar plot showing the distribution of cells in G1, S and G2-M phases in A2780 cell line transiently expressing Drp1shRNA1/2 as normalized by the distribution of A2780 cell line transiently expressing a non-targeting (NT) shRNA. **D.** Immunoblot showing lentiviral mediated transient knockdown of Drp1 protein levels using two distinct Drp1 shRNAs as compared to that of a non-targeting (NT) shRNA in SKOV3 cell line. Actin is used as a loading control. **E.** Bar plot showing the distribution of cells in G1, S and G2-M phases in SKOV3 cell line transiently expressing Drp1shRNA2 as normalized by the distribution of SKOV3 cell line transiently expressing a non-targeting (NT) shRNA. **F.** Bar plot depicting cell proliferation of A2780 and SKOV3 cell lines transiently expressing Drp1 shRNA1/2 and NT shRNA.

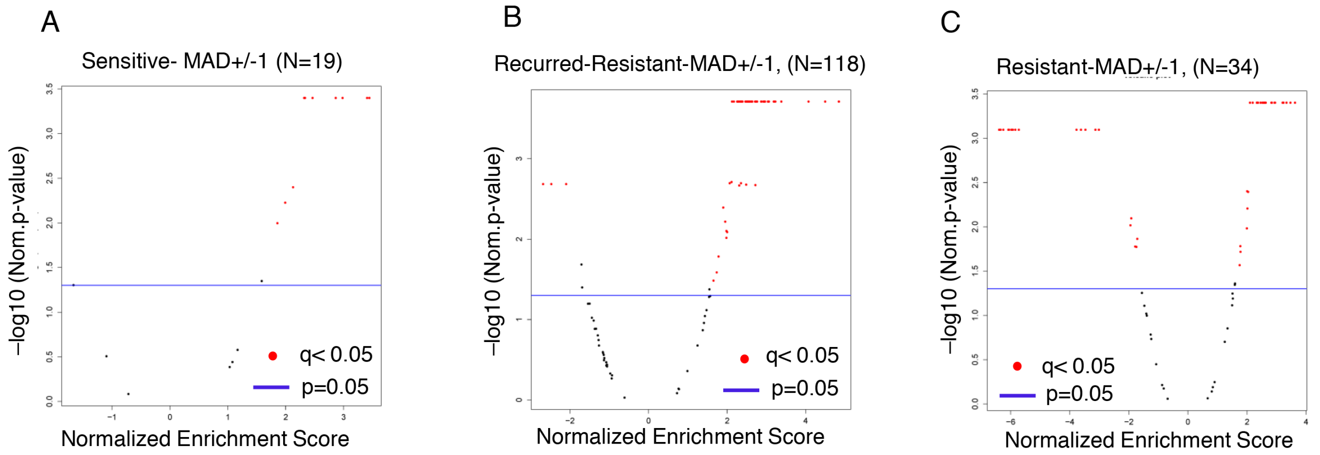

Supplementary Figure S8: A. Volcano plot representing the output of the GSEA analyses of statistically significant Drp1 correlated genes in the TCGA EOC sensitive patients. **B.** Volcano plot representing the output of the GSEA analyses of statistically significant Drp1 correlated genes in the TCGA EOC recurred-resistant patients. **C.** Volcano plot representing the output of the GSEA analyses of statistically significant Drp1 correlated genes in the resistant TCGA EOC patients. Negatively and positively correlated pathways have negative and positive Normalized Enrichment Scores respectively. N depicts number of patients.

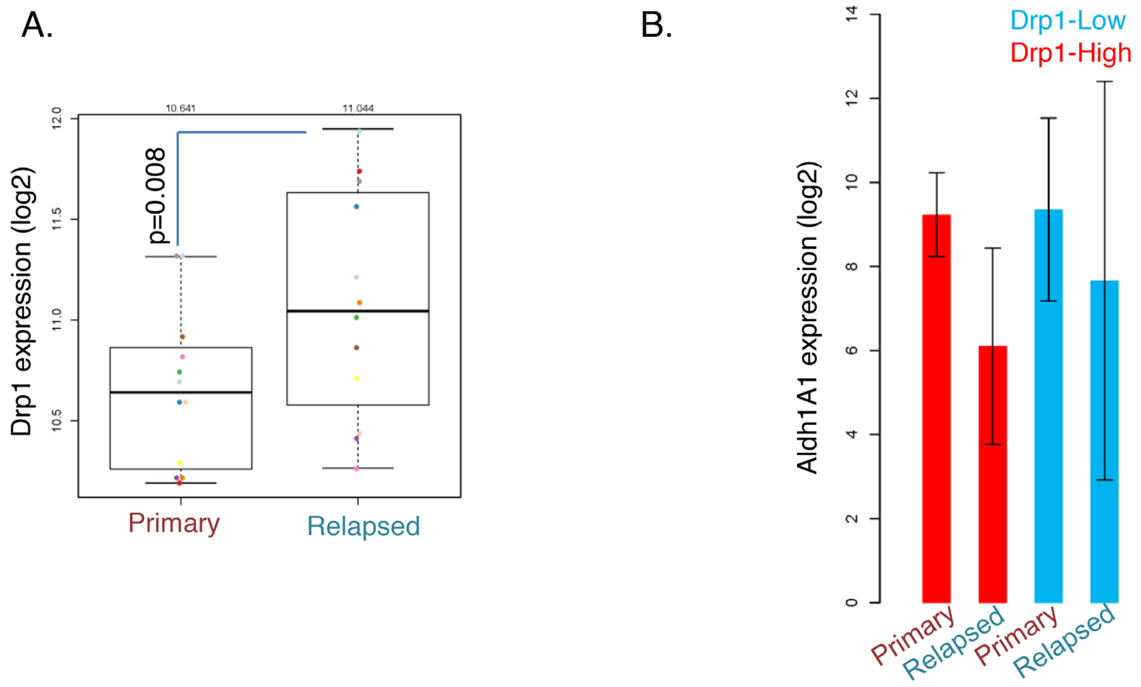

Supplementary Figure S9: A. Box plot of Drp1 expression in the primary and relapsed samples of recurred-resistant EOC patient cohort of ICGC. Median values of Drp1 expression mentioned on the top. **B.** Barplots showing mean Aldh1A1 expression in the primary and relapsed samples of Drp1-High and Drp1-Low patient groups of ICGC.

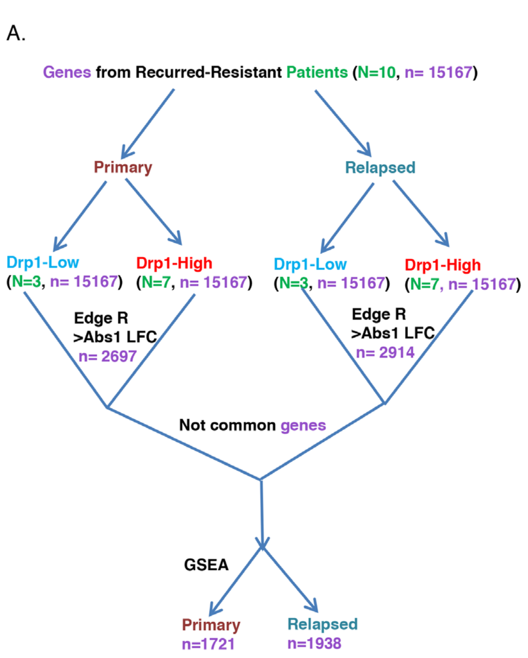

Supplementary Figure S10: A. Scheme to identify the pathways that are differentially regulated uniquely in the primary or in the relapsed samples between the Drp1-High and Drp1-Low groups. **B.** Scheme to identify the pathways that are differentially regulated uniquely in the Drp1-High or Drp1-Low groups between their respective primary and relapsed samples. Abs1 LFC : Absolute 1 Log fold change in expression.

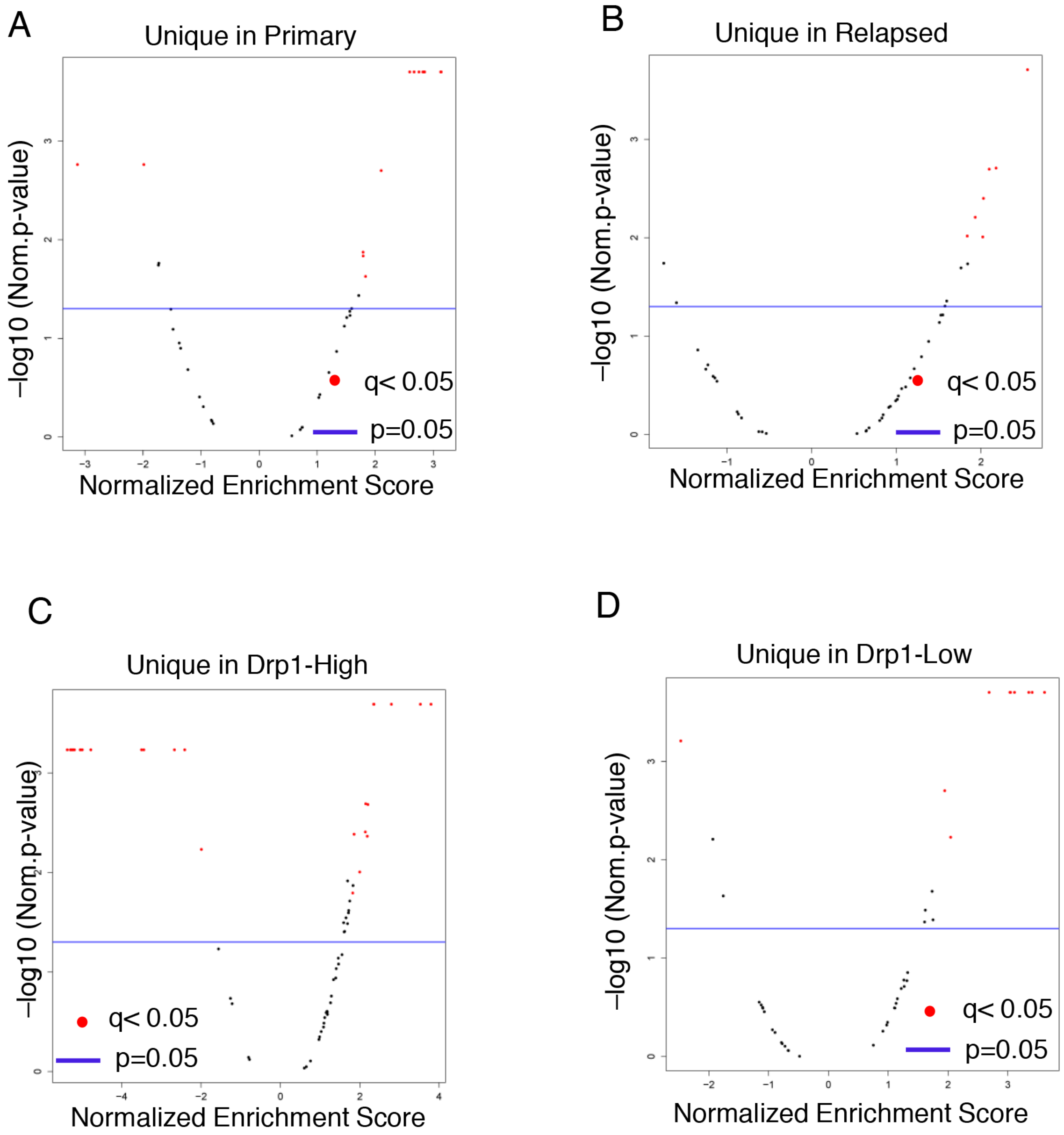

Supplementary Figure S11: A. Volcano plot representing the output of the GSEA analyses of genes that are differentially expressed uniquely in the primary samples between the ICGC Drp1-High and Drp1-Low groups. **B.** Volcano plot representing the output of the GSEA analyses of genes that are differentially expressed uniquely in the relapsed samples between the ICGC Drp1-High and Drp1-Low groups. **C.** Volcano plot representing the output of the GSEA analyses of genes that are differentially expressed uniquely in the Drp1-High group between the primary and the relapsed samples. **D.** Volcano plot representing the output of the GSEA analyses of genes that are differentially expressed uniquely in the Drp1-Low group between the primary and the relapsed samples. Negatively and positively correlated pathways have negative and positive Normalized Enrichment Scores respectively.

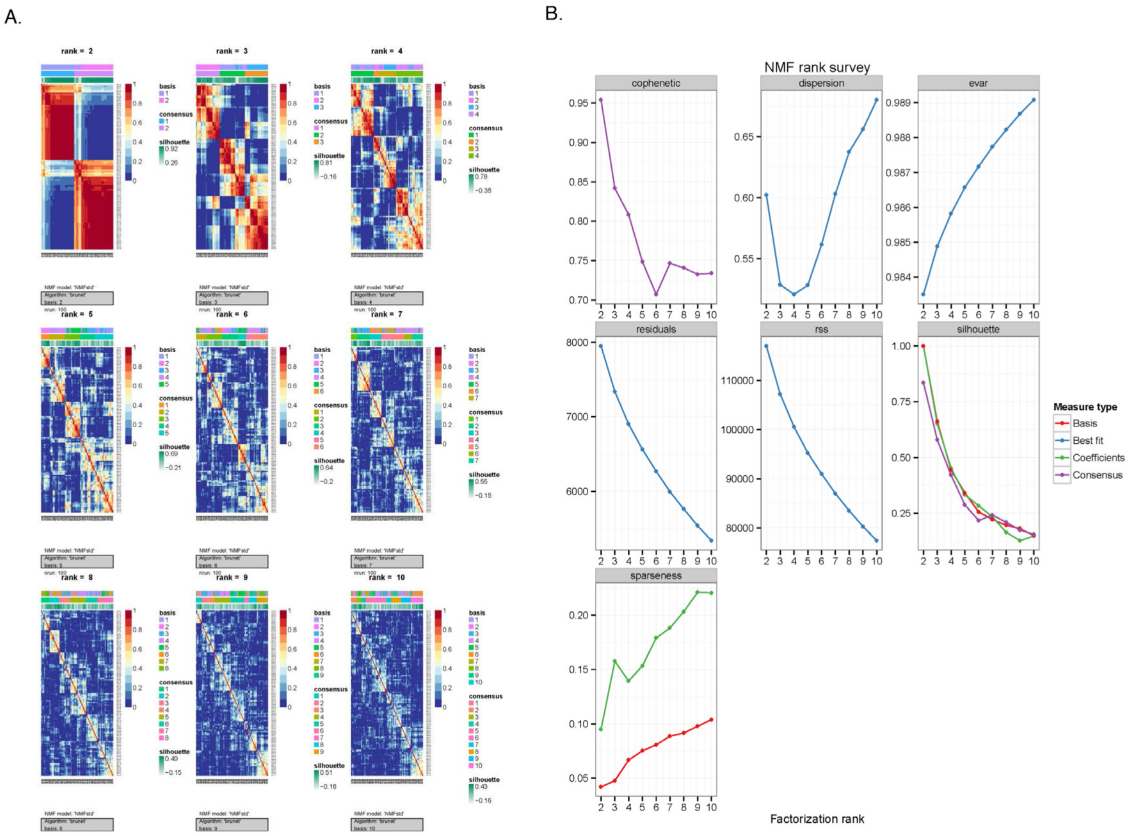

Supplementary Figure S12: A. Heatmaps created by the R NMF package showing the consensus clusters created by NMF with rank 2 to 10 with rank 2 being the most stable cluster. **B.** NMF quality measures computed from NMF runs for each value of rank (2 to 10) with clusters corresponding to rank 2 having the highest silhouette and cophenetic score.

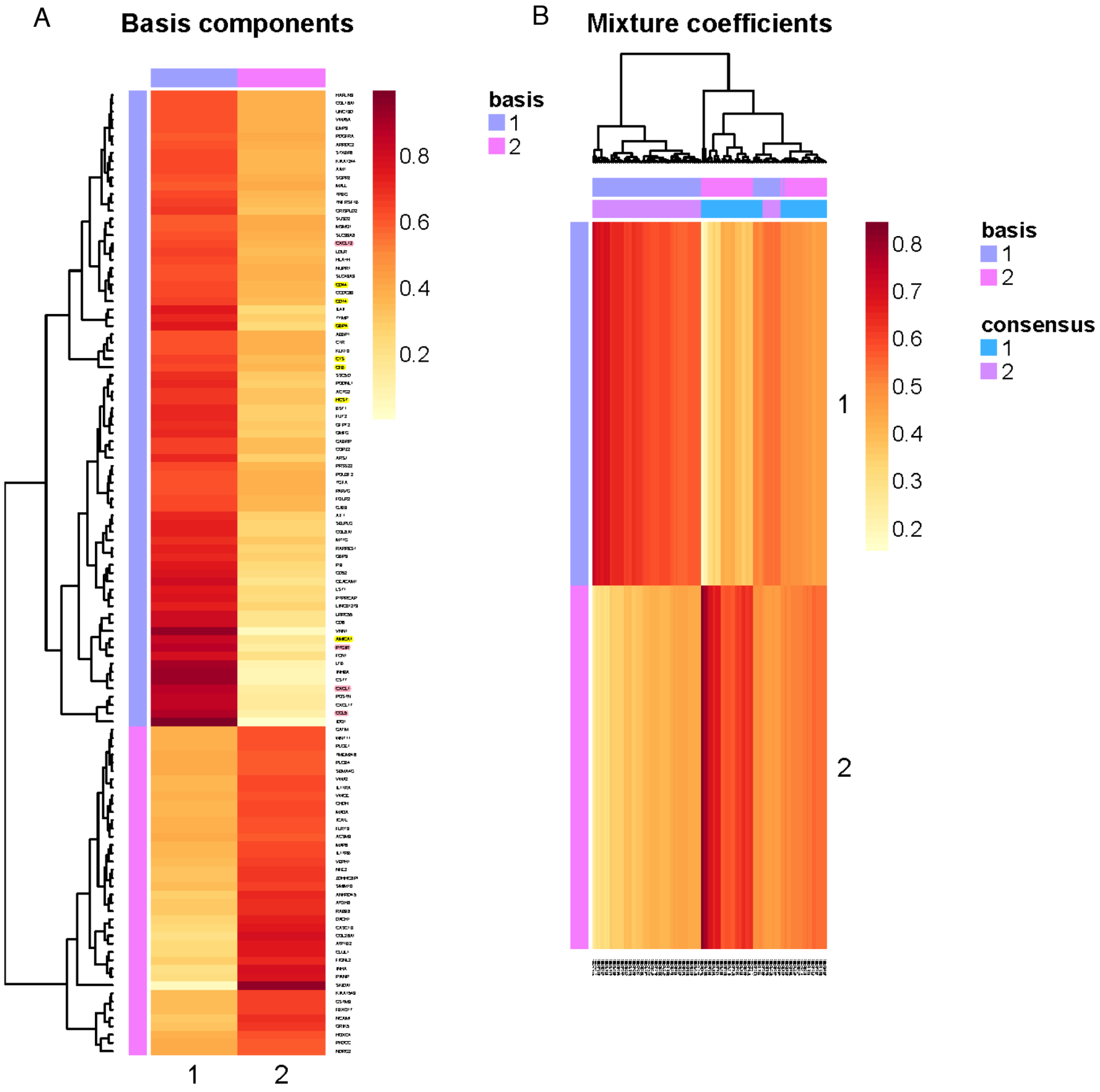

Supplementary Figure S13: A. Heatmap of the basis component matrix for rank 2 as determined by Supplementary Fig 12. The rows represent genes and two columns represent two metagenes. The cells are color-coded representing the weight of each gene in each metagene. The genes were clustered into two metagenes using hierarchical clustering. **B.** Heatmap of mixture coefficients matrix (metasamples) for rank 2. The two rows represent two metasamples and the columns represent the original samples. The samples were clustered into two metasamples based on the expression of the two metagenes. The cells are color-coded based on the weight of the each sample in each metasample.

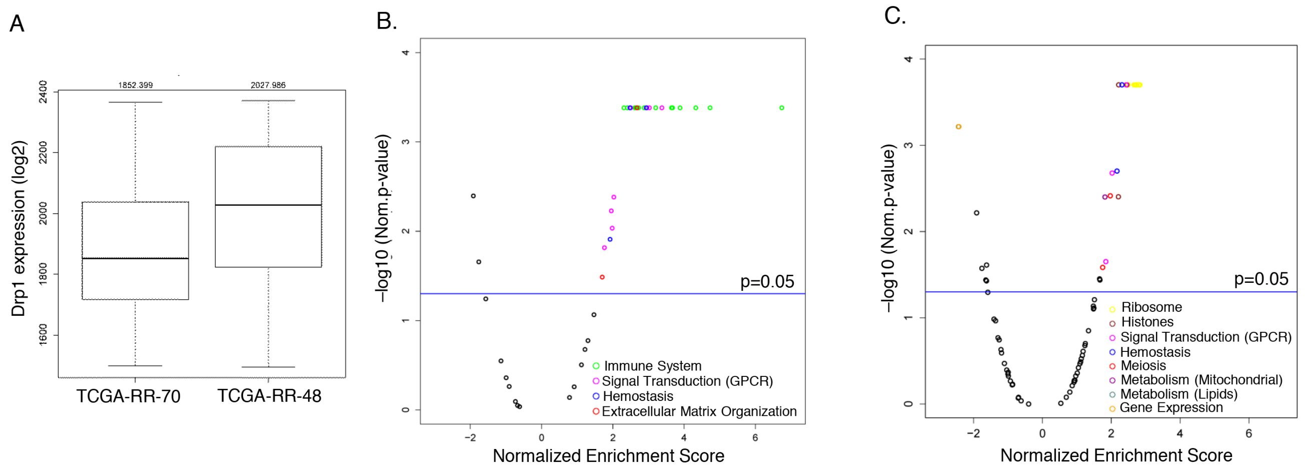

Supplementary Figure S14: A. Box plot of Drp1 expression in the primary samples of recurred-resistant EOC patient cohort of TCGA that was classified as TCGA-RR-70 and TCGA-RR-48 based on a Drp1 –based-gene-expression-signature. Median values of Drp1 expression mentioned on the top. **B.** Volcano plot representing the output of the GSEA analyses of genes that are differentially expressed between the TCGA-RR-70 and TCGA-RR-48 patient groups. Negatively and positively correlated pathways have negative and positive Normalized Enrichment Scores respectively. N depicts number of patients. **C.** Volcano plot representing the output of the GSEA analyses of genes that are differentially expressed between the ICGC-Drp1-High and ICGC-Drp1-Low patient groups. Negatively and positive correlated pathways have negative and positive Normalized Enrichment Scores respectively.

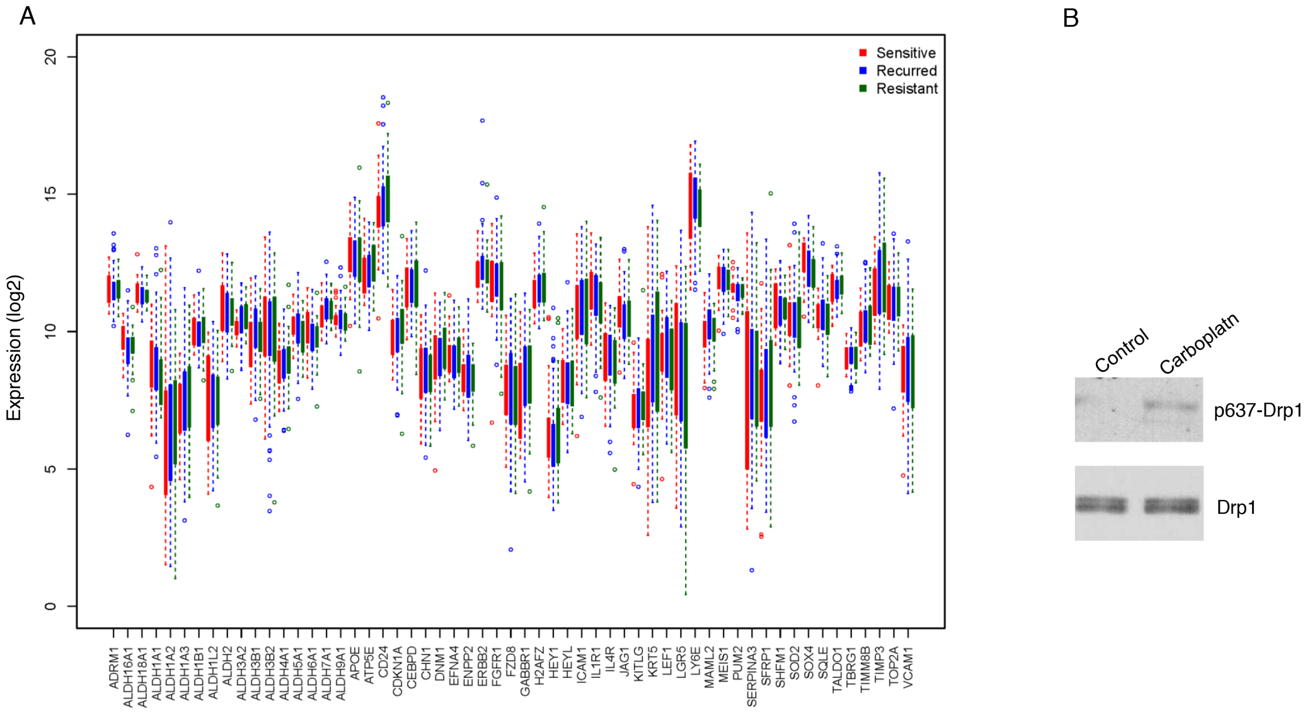

Supplementary Figure S15: A. Box plot of CSC gene signature in the primary tumor tissues of sensitive, recurred-resistant and resistant EOC patients. **B.** Immunoblot analysis of impact of carboplatin (5μM, 24 hours) on Drp1 phosphorylation with total Drp1 as control.

Supplementary Table S1: A. TCGA Cancer types with sample counts and DNM1L expression values. **B.** ICGC samples information. **C.** ICGC samples paired (Primary-Relapsed)

See Supplementary File 1

Supplementary Table S2: A. Summary of GSEA analyses of Drp1 correlating genes in ACC **B.** Summary of GSEA analyses of Drp1 correlating genes in UVM. **C.** Summary of GSEA analyses of Drp1 correlating genes in UCS. **D.** Summary of GSEA analyses of Drp1 correlating genes in UCEC. **E.** Summary of GSEA analyses of Drp1 correlating genes in THYM. **F.** Summary of GSEA analyses of Drp1 correlating genes in THCA. **G.** Summary of GSEA analyses of Drp1 correlating genes in TCGT. **H.** Summary of GSEA analyses of Drp1 correlating genes in SKCM. **I.** Summary of GSEA analyses of Drp1 correlating genes in SARC. **J.** Summary of GSEA analyses of Drp1 correlating genes in READ. **K.** Summary of GSEA analyses of Drp1 correlating genes in PRAD. **L.** Summary of GSEA analyses of Drp1 correlating genes in PCPG. **M.** Summary of GSEA analyses of Drp1 correlating genes in PAAD. **N.** Summary of GSEA analyses of Drp1 correlating genes in OV. **O.** Summary of GSEA analyses of Drp1 correlating genes in MESO. **P.** Summary of GSEA analyses of Drp1 correlating genes in LUSC. **Q.** Summary of GSEA analyses of Drp1 correlating genes in LUAD. **R.** Summary of GSEA analyses of Drp1 correlating genes in LIHC. **S.** Summary of GSEA analyses of Drp1 correlating genes in LGG. **T.** Summary of GSEA analyses of Drp1 correlating genes in LAML. **U.** Summary of GSEA analyses of Drp1 correlating genes in KIRP. **V.** Summary of GSEA analyses of Drp1 correlating genes in KIRC. **W.** Summary of GSEA analyses of Drp1 correlating genes in KICH. **X.** Summary of GSEA analyses of Drp1 correlating genes in HNSC. **Y.** Summary of GSEA analyses of Drp1 correlating genes in GBM. **Z.** Summary of GSEA analyses of Drp1 correlating genes in DLBC. **AA.** Summary of GSEA analyses of Drp1 correlating genes in COAD. **AB.** Summary of GSEA analyses of Drp1 correlating genes in CHOL. **AC.** Summary of GSEA analyses of Drp1 correlating genes in CESC. **AD.** Summary of GSEA analyses of Drp1 correlating genes in BRCA. **AE.** Summary of GSEA analyses of Drp1 correlating genes in BLCA. AC AC

See Supplementary File 2

Supplementary Table S3: A. Drp1 Correlation with all other genes which do not have any 0 as expression values in the Drp1-MAD1 group. **B.** Drp1 Correlation with all other genes which do not have any 0 as expression values in the Drp1-MAD1-Gistic-0 group

See Supplementary File 3

Supplementary Table S4: A. Summary of GSEA analyses of Drp1 co-expressing genes in EOC patients wihtin Drp1-MAD1 group using REACTOME. **B.** Summary of GSEA analyses of Drp1 co-expressing genes in EOC patients within Drp1-MAD1 group using KEGG. **C.** Summary of GSEA analyses of Drp1 co-expressing genes in EOC patients within Drp1-MAD1-Gistic-0 group using REACTOME.

See Supplementary File 4

Supplementary Table S5: Top level Reactome categpries categories positively (up) or negatively (down) correlating with Drp1 expression

See Supplementary File 5

Supplementary Table S6: A. Summary of GSEA analyses of FIS1 co-expressing genes in EOC patients wihtin FIS1-Gistic0 group. **B.** Summary of GSEA analyses of MFF co-expressing genes in EOC patients within MFF-Gistic0 group. **C.** Summary of GSEA analyses of MFN2 co-expressing genes in EOC patients within MFN2-Gistic0 group. **D.** Summary of GSEA analyses of DNM1L-FIS1 co-expressing genes in EOC patients within DNM1L-FIS1-Gistic0 group. **E.** Summary of GSEA analyses of DNM1L-MFF co-expressing genes in EOC patients within DNM1L-MFF-Gistic0 group. **F.** Summary of GSEA analyses of DNM1L-MFN2 co-expressing genes in EOC patients within DNM1L-MFN2-Gistic0 group.

See Supplementary File 6

Supplementary Table S7: A. Leading edge genes of the Drp1:Mff overlapping Cell Cycle pathways present in Drp1:Fis1 gene overalp. **B.** Leading edge genes of the Drp1:Mff overlapping Cell Cycle pathways present in Drp1:Mff gene overlap. **C.** Leading edge genes of the Drp1:Mff overlapping Cell Cycle pathways present in Drp1:Mfn1 gene overlap. **D.** Leading edge genes of the Drp1:Mff overlapping Cell Cycle pathways present in Drp1:Mfn2 gene overlap. **E.** Leading edge genes of the Drp1:Mff overlapping Cell Cycle pathways present in Drp1:Opa1 gene overlap.

See Supplementary File 7

Supplementary Table S8: A. Summary of GSEA analyses of Drp1 co-expressing genes in sensitive EOC patients within Drp1-MAD1 group. **B.** Summary of GSEA analyses of Drp1 co-expressing genes in recurred-resistant EOC patients within Drp1-MAD1 group. **C.** Summary of GSEA analyses of Drp1 co-expressing genes in resistant EOC patients within Drp1-MAD1 group.

See Supplementary File 8

Supplementary Table S9: A. Summary of GSEA analyses of genes differentially expressed uniquely in the primary samples between the Drp1-High and Drp1-Low groups. **B.** Summary of GSEA analyses of genes differentially expressed uniquely in the ascites samples between the Drp1-High and Drp1-Low groups. **C.** Summary of GSEA analyses of genes differentially expressed uniquely in the Drp1-High group between the primary and ascites samples. **D.** Summary of GSEA analyses of genes differentially expressed uniquely in the Drp1-Low group between the primary and ascites samples.

See Supplementary File 9

Supplementary Table S10: A. NMF clustering of the TCGA recurred-resistant EOC patients. **B.** NMF asignments of the genes of the “Drp1 based gene expression signature” in the classified TCGA recurred-resistant cohort.

See Supplementary File 10
